# Production of Mesenchymal Stem Cells through Stem Cell Reprogramming

**DOI:** 10.3390/ijms20081922

**Published:** 2019-04-18

**Authors:** Ahmed Abdal Dayem, Soo Bin Lee, Kyeongseok Kim, Kyung Min Lim, Tak-il Jeon, Jaekwon Seok, Ssang-Goo Cho

**Affiliations:** Department of Stem Cell & Regenerative Biotechnology, Incurable Disease Animal Model and Stem Cell Institute (IDASI), Konkuk University, Gwangjin-gu, Seoul 05029, Korea; ahmed_morsy86@yahoo.com (A.A.D.); soobineey@naver.com (S.B.L.); proproggs@naver.com (K.K.); lmin0217@naver.com (K.M.L.); jeonti94@naver.com (T.-i.J.); tjrwornjs@naver.com (J.S.)

**Keywords:** pluripotent stem cells (PSCs), mesenchymal stem cells (MSCs), tissue-derived mesenchymal stem cells, pluripotent stem cells-derived mesenchymal stem cells (PSC-MSCs), differentiation methods, in vitro and in vivo therapeutic efficacies

## Abstract

Mesenchymal stem cells (MSCs) possess a broad spectrum of therapeutic applications and have been used in clinical trials. MSCs are mainly retrieved from adult or fetal tissues. However, there are many obstacles with the use of tissue-derived MSCs, such as shortages of tissue sources, difficult and invasive retrieval methods, cell population heterogeneity, low purity, cell senescence, and loss of pluripotency and proliferative capacities over continuous passages. Therefore, other methods to obtain high-quality MSCs need to be developed to overcome the limitations of tissue-derived MSCs. Pluripotent stem cells (PSCs), including embryonic stem cells (ESCs) and induced pluripotent stem cells (iPSCs), are considered potent sources for the derivation of MSCs. PSC-derived MSCs (PSC-MSCs) may surpass tissue-derived MSCs in proliferation capacity, immunomodulatory activity, and in vivo therapeutic applications. In this review, we will discuss basic as well as recent protocols for the production of PSC-MSCs and their in vitro and in vivo therapeutic efficacies. A better understanding of the current advances in the production of PSC-MSCs will inspire scientists to devise more efficient differentiation methods that will be a breakthrough in the clinical application of PSC-MSCs.

## 1. Introduction

**Figure 1:**
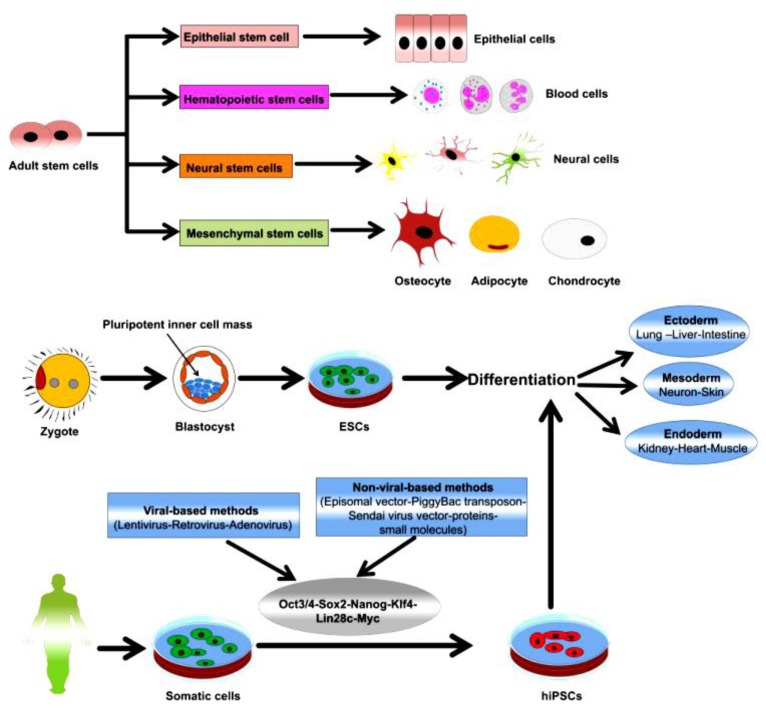
Schematic diagram outlining the classes of stem cells and their differentiation capacities. Reproduced from article by Abdal Dayem et al. 2018 [5], which is an open access article distributed under the terms and conditions of the Creative Commons Attribution (CC BY) license (http://creativecommons.org/licenses/by/4.0/).

Stem cells are mainly classified as adult stem cells (ASCs), embryonic stem cells (ESCs), and induced pluripotent stem cells (iPSCs) (Figure 1). Mesenchymal stem cells (MSCs) are considered as the main class of ASCs with prominent therapeutic efficacies. MSCs are multipotent stem cells that can be easily isolated from various tissues and organs of the human body, such as bone, fat tissue, cartilage, hepatic tissue, blood, and muscle [1,2,3,4].

In 1959, bone marrow (BM) transplant, as a hematopoietic stem cell-based therapy, was applied for the first time in patients after confirming its therapeutic effects in dogs [6]. MSCs were first identified by Alexander Friedenstein as a colony-forming unit fibroblast and osteoprogenitor with a fibroblast-like shape, which grows in cell colonies and adheres to the culture plate, differing from the hematopoietic stem cell (HSC) [7]. MSCs possess a wide range of therapeutic applications [8,9]. In 2007, Sacchetti et al. proved the self-renewal potential of osteoprogenitors in BM sinusoids by showing their capacity to organize the hematopoietic microenvironment [10]. In this study, they showed the capacity of melanoma cell-adhesion molecule (MCAM)/cluster of differentiation 146 (CD146)-expressing subendothelial cells located in the stroma of BM to transfer the hematopoietic microenvironment to heterotopic sites and to simultaneously form subendothelial cells in miniature bone organs upon transplantation.

The application of MSCs obviates critical concerns emerging from the use of PSCs, such as ethical issues, histocompatibility concerns, and tumorigenicity [11,12,13,14]. The therapeutic capacity of MSCs is mostly dependent on its potential to migrate and home to the damaged tissue or via secretion of various bioactive factors and molecules with therapeutic activities (paracrine or hit-and-run action) [15,16,17]. The potent immunomodulatory functions of MSCs allow them to be applied in the alleviation of graft-versus-host disease and in various clinical trials for disease therapy [18,19]. Moreover, the capacity of these cells to differentiate into multi-lineages, such as chondrogenic, osteogenic, and adipogenic differentiation, has been shown.

The quality of MSCs can markedly change based on the cell source, media composition, and cell passage, which is reflected as alterations in cell morphology, DNA abnormalities, as well as cell senescence, decline in the proliferation and differentiation capacity, and changes in cellular plasticity [20,21,22,23,24]. Of note, variations in the quality of MSCs can influence reproducibility, creating inconsistencies in the in vivo findings and, ultimately, clinical trials [25,26,27,28]. In addition, there are many issues that can occur in tissue-derived stem cells, including environmentally or genetically mediated DNA abnormalities that possess accumulative effects over the passaging time to markedly influence the quality and longevity of stem cells for further application [22,29]. Moreover, isolation of BM-derived MSCs (BM-MSCs) requires an invasive and painful surgical procedure, and the capacity of these cells to proliferate and differentiate markedly declines over long passages [30,31]. To overcome these hurdles, scientific interest is directed toward devising an efficient culture system to generate high-quality MSCs for reproducible results in regenerative medicine. Among these methods, the derivation of MSCs from PSCs is one crucial alternative. PSC-derived MSCs (PSC-MSCs) possess in vitro and in vivo multi-differentiation potential and immunomodulation functions as shown by the primary MSCs [32]. PSC-MSCs can be produced at a large scale with higher purity and with more efficient genetic manipulation compared with tissue-derived MSCs [33].

In this review, we will provide an overview on the sources, retrieval methods, and characterization of MSCs, showing how these factors influence the quality and further clinical applications of MSCs. Moreover, we will describe in detail possible methods for the derivation of MSCs from PSCs, with an emphasis on the merits and disadvantages of each method and possible recommendations for further improvement. Finally, the hurdles and challenges of MSC generation will be discussed. Thus, large-scale production of high-quality PSC-MSCs that efficiently proliferate and differentiate with high reproducibility can be a powerful tool for regenerative medicine.

## 2. Overview of MSCs

### 2.1. MSC Sources

MSCs were originally identified in BM and described as multi-potent stromal precursor cells with fibroblast-like morphology forming colonies; therefore, they have been identified as colony-forming unit fibroblasts (CFU-F) [34,35,36]. Further, there are various sources to obtain MSCs other than BM, including muscle tissue [37], fat tissue [38], periodontal ligament [39], peripheral blood [40], synovial fluid, salivary gland [41], alveolar epithelium [42], umbilical cord [43], and dental pulp [44]. Adipose-derived MSCs (AD-MSCs) are easily isolated and are considered a vital source of MSCs for tissue regeneration [45]. Moreover, fetal tissues, in particular umbilical cord tissues, are rich sources of MSCs [46,47,48,49,50]. The molecular characteristics, surface antigen expression, and biological functions such as proliferation and differentiation capacities of MSCs can vary based on the MSC source [46,51,52,53,54].

MSCs can be autologous (same patient-derived) or allogeneic (derived from another patient), which differentially influences the clinical application of MSCs [55,56,57,58]. Allogeneic MSCs are more applicable in clinical trials than autologous MSCs [55,59]. Owing to the advantageous immunogenic characteristics and superior clinical applications of allogeneic MSCs, they are called “universal donor cells” [56,60].

### 2.2. MSC Characterization

In 2006, the International Society for Cellular Therapy (ISCT) proposed basic criteria to characterize MSCs [61]. In brief, isolated MSCs must efficiently adhere to the plastic culture vessel under optimal culture conditions. In addition, MSCs were identified to express specific surface markers, which vary according to the tissue of origin [62,63]. MSCs positively express CD105 (endoglin), CD90 (Thy-1), CD166, CD44, and CD73 (lymphocyte-vascular adhesion protein 2), whereas they negatively express human leukocyte antigen (HLA-DR), CD14 (a co-receptor for the bacterial lipopolysaccharide detection), CD34 (hematopoietic stem cells and endothelial cells markers), CD19 (B cell antigen), CD79a, CD11b (integrin α M), and CD45 (leukocyte marker) [61,64,65,66,67]. Additional markers can also be expressed in MSCs, namely, stage-specific embryonic antigen-4 (SSEA-4) [68], MSC antigen 1 (MSCA-A) [69], STRO-1 [70,71], CD271/NGFR [72,73], and CD146 [10]. STRO-1 is a definite marker in BM-MSCs, particularly in cells during early passages, and its expression decreases as the cell is passaged [71,74,75]. Accordingly, STRO-1-expressing MSCs are considered suitable cells for medical translation [76]. Positive expression of CD271 in BM-MSCs denotes its multi-potency [77]. Neural stem cell marker, nestin, is a specific marker expressed in BM-MSCs [78,79]. Further, an interesting study showed that BM-MSCs with high expression of platelet-derived growth factor receptor alpha (PDGFRα) and STRO-1 have high growth rates and a high capacity for in vivo bone formation compared with cells with low expression of these markers [80]. Of note, this study is based on BM-MSCs isolated from a wide range of donors. The surface markers of MSCs can be analyzed with fluorescence-activated cell sorting (FACS) analysis. Although the expression of the aforementioned surface markers cannot be a complete verification of the purity and homogeneity of isolated MSCs [81], specific surface markers of MSCs can provide powerful evidence about the proliferation, homogeneity, and differentiation capacity of MSCs.

Besides the expression of surface markers, MSCs can be characterized by self-renewability, multi-potency, and multi-differentiation capacity [10,81], and the differentiation capacity of MSCs into various lineages, such as osteocytes, adipocytes, chondrocytes, heart cells, neurons, and myocytes, has been shown in previous reports [82,83,84,85,86,87].

## 3. Derivation of MSCs from PSCs: Methods and Applications

### 3.1. MSCs Derived from ESCs

#### 3.1.1. Basic Methods

The derivation of MSCs from PSCs was originally employed for the first time in ESCs, and so here we will describe the basic methods for the production of ESC-MSCs. In 2004, Xu et al. carried out the first trial to generate MSC-like cells from human ESCs (hESCs) [88]. In this study, hESC was differentiated into a fibroblast-like cell via the dissociation of hESCs into aggregates cultured in a non-adherent culture plate using differentiation media composed of knockout Dulbecco’s modified Eagle’s medium (KO-DMEM), 20% fetal bovine serum (FBS), β-mercaptoethanol, L-glutamine, and 1% nonessential amino acids (NEAA) for four days. To increase the immortalization of the derived cells, ectopic expression of human telomerase reverse transcriptase (hTERT) using a retrovirus system was performed. The resultant cells possessed MSC-like characteristics confirmed by surface marker expression, alkaline phosphatase (ALP) activity, and the osteogenic differentiation capacity.

In 2005, another research group devised a simple protocol to generate multipotent mesenchymal precursors from undifferentiated hESCs through the co-culture of two hESCs, namely H1 and H9 hESCs, with the mouse BM stroma cell line, OP9, to enhance mesodermal differentiation [89]. This differentiation was carried out using α minimum essential medium (MEM) supplemented with 20% FBS for 40 days; then, the differentiated cells were sorted for expression of the adult MSC surface marker, CD73. In this study, 5% of the differentiated cells positively expressed CD73 and was re-cultured onto a cell culture plate using αMEM supplemented with 20% FBS for one to two weeks. FACS analysis of CD73-positive cells showed expression of STRO-1, CD105, CD106, CD29, CD54, CD44, and CD166 and lack of expression of CD14, CD34, and CD45.

In 2006, Olivier et al. developed a reproducible method for the generation of MSCs from hESCs that avoided the use of animal-based feeder cells to produce clinical-grade MSCs [90]. This method is based on the mechanical removal of spontaneously differentiated cells called raclures or scraped cells, which are located at the periphery or the center of hESC colonies. Following this, these raclures were plated again using D10 medium with 10% FBS, and thick epithelial cells were observed after one month of culture. These cells showed morphological and molecular characteristics similar to those of BM-MSCs and could be maintained for 20–25 passages. This method is based on the mechanical picking of spontaneously differentiated cells based on visual evaluation that ultimately leads to the generation of a heterogonous cell population containing non-MSCs, which is considered a critical disadvantage of this method.

In 2007, Trivedi et al. performed a co-culture of the H1 and H9 hESC lines with the irradiated OP9 cells to generate MSCs [91], and this method was similar to the study employed by Barberi et al. [89]. However, the authors could obtain CD73^+^ and CD34^+^ (primitive hematopoietic cells marker) cells earlier (within two weeks) than that detected by Barberi et al. (at day 40). The generated CD34^+^ cells could produce hematopoietic progenitor colonies. The authors attributed the emergence of CD34^+^ cells to the culture of the undifferentiated hESCs in Matrigel-coated plate in mouse embryonic fibroblast (MEF)-conditioned medium (CM) for several passages instead of maintaining on MEF before the start of differentiation process. In 2008, Trivedi et al. devised a method for the differentiation of MSCs from ESCs without co-culture with OP9 cells, which was based on plating hESC lines onto Matrigel plates in MEF-CM supplemented with basic fibroblast growth factors (bFGF) and passaged several times [92]. The differentiated cells showed positive expression of CD73 and negatively expressed SSEA-4 and resembled BM-MSCs in the morphology, surface characteristics, and tri-lineage differentiation capacity [92]. Moreover, these cells expressed HLA class I, whereas they lacked the expression of class II HLA. Using mixed lymphocyte reaction assays, hESC-MSCs inhibited the proliferation of responder T-lymphocytes [92].

In 2008, Hwang et al. attempted to generate MSCs form hESCs using differentiated embryoid bodies (EBs) [93]. In this method, hESCs differentiated into EBs for 10 days, and the formed EBs were plated onto a gelatin-coated culture plate and further cultured for 10 days until the sprawling of fibroblast-like cells from the EBs adhered to the plate. These adhered cells were collected by mechanical scraping and, at passage 7, the differentiated cells expressed MSC-surface markers. The derived hESC-MSCs were then subjected to the chondrogenic differentiation through exposure to primary bovine chondrocyte-CM. Subsequently, the authors tested the in vivo commitment of the hESC-MSC-expanded in chondrocyte-CM. For this purpose, these cells were encapsulated in PEG-diacrylate (PEGD) hydrogel and transplanted into athymic mice and the CM of these cells was transplanted into athymic nude rats with articular cartilage defects [93]. The transplantation of encapsulated cells and the transplanted CM resulted in ectopic extracellular matrix (ECM) rich-cartilage formation and the complete recovery of the cartilage defects, respectively.

In 2009, Brown et al. attempted to generate hESC-derived MSCs, which were generated from EB clusters [94]. EB formation was carried out through the culturing of hESCs in a petri dish (with low-attachment) with hESC culture media. The formed EBs were plated onto a gelatin-coated plate and cultured with human BM-MSC (hBM-MSC) culture media. The generated cells showed normal karyotype and characteristic features similar to those of BM-MSCs, such as positive expression of STRO-1 and CD73.

#### 3.1.2. MSCs Derived via Repeated Passages Using Trypsinization with MSC Culture Medium

In 2011, Yen et al. derived mesenchymal progenitors (MPs) from hESC with a simple procedure that excluded EB preparation, using feeder cells and cell sorting [95]. This method was based on shifting from hESC medium to BM-MSC culture medium. Upon reaching confluency, the cells were dissociated with 0.025% trypsin/Ethylenediaminetetraacetic acid (EDTA. The cell characterization was based on transcriptome profiling using gene expression microarray. hESC-MPs expressed similar markers to BM-MSCs, which were positive in expression of CD29, CD44, CD73, CD90, and CD105 and negative in expression of the hematopoietic markers (CD14, CD34, and CD45). Moreover, they showed expression of the neural stem cell marker, nestin, but lacked ALP activity. Of note, weak expression of hESCs markers, such as SSEA-4 and CD9, was detected [95]. However, the other hESC markers, including TRA-1-60 and TRA-1-81, were not expressed. hESC-derived MPs were negative in the expression of MHC class II molecules, including HLA-DR, whereas weak expression of class I molecule and the non-classical MHC I molecule (HLA-G) was shown. In contrast, BM-MSCs showed high expression of MHC class II molecules.

Compared with BM-MSCs, hESC-MPs possessed similar tri-lineage differentiation capacity but showed a markedly higher proliferative capacity without alteration in the cell karyotype [95]. The subcutaneous (s.c.) injection of hESC-MPs in immune-compromised mice (NOD/severely combined immunodeficient (SCID) mice) did not show any teratoma formation until four months. Taken together, hESC-MPs possessed unique features compared with BM-MSCs, and the therapeutic applications of these cells need to be investigated in further studies.

#### 3.1.3. Hemangioblast-Based Methods

The previously described methods are considered laborious and depend on scraping, handpicking, and sorting. Other research groups attempted to prepare PSC-MSCs through differentiation of ESCs into bi-potential progenitors called hemangioblasts. Hemangioblasts are intermediate precursors that connect differentiation between PSCs and MSCs, allow the initial PSC differentiation to be directed rather than random, and eliminate transfer of any remaining pluripotent cells from the original PSCs [26]. The methods for hemangioblast generation from hESCs are described in previous reports [96,97].

Kimbrel et al. generated MSCs from hESCs via hemangioblast generation as intermediate cells [26]. To generate hemangioblast, hESCs were initially differentiated into EBs for four days via culture onto a low-adherence culture dish using Stemline II medium supplemented with vascular endothelial growth factor (VEGF), bFGF, and bone morphogenetic protein 4 (BMP4). Subsequently, the differentiated EBs were trypsinized and cultured under complete serum-free methylcellulose-based medium supplemented with plenty of cytokines for three to four days, which resulted in the formation of shiny aggregations of spherical hemangioblasts [97]. These cells were allowed to expand further for 9–10 days. For MSC differentiation, the expanded hemangioblasts were cultured in αMEM supplemented with 20% serum onto a Matrigel-coated culture plate to differentiate into MSCs (passage 0). Next, fibroblast-like cells began to adhere to the culture plate and were incubated for five days. These attached cells were trypsinized and cultured for further differentiation into MSCs of passage 1 with the removal of non-adherent cells. Afterwards, the cells were continuously cultured until high confluency (70%–80%) was obtained and then characterized [26]. The differentiated hESC-MSCs showed a similar immune-phenotype to that of BM-MSCs isolated from various donors. However, hESC-MSCs showed higher proliferative capacity (twice), smaller cell size, lower expression of STRO-1, and higher expression of CD10 and CD24 than that of the isolated BM-MSCs [26]. Of note, these differentiated hESC-MSCs showed potent therapeutic capacity against autoimmune disease mouse models, namely experimental autoimmune encephalitis (EAE) mouse and lupus nephritis and uveitis.

In 2014, Wang et al. confirmed the hemangioblast-mediated MSC differentiation procedure performed by Kimbrel et al. [98]. Interestingly, in the animal study with the EAE mouse model, the therapeutic effect of hESC-MSCs surpassed that of BM-MSCs isolated from different donors, showing better protective action against neuronal demyelination. The potent anti-inflammatory action of hESC-MSCs against EAE is attributed to lower interleukin-6 (IL-6) expression compared with its expression in donor-isolated BM-MSCs [98]. Moreover, hESC-MSCs showed a higher migratory capacity into the inflamed neuronal tissue via extravasation of the blood–brain barrier and blood–spinal cord barrier than that shown by normal BM-MSCs.

Collectively, hESC-MSCs showed superior therapeutic capacity against autoimmune inflammatory disorders than BM-MSCs isolated from various donors, which is ascribed to the unique characteristics of hESC-MSCs. However, further in-depth studies to delve into the detailed molecular characteristics and key therapeutic molecules of hESC-MSCs are needed.

#### 3.1.4. Defined Culture-Based Methods

The aforementioned methods relied mainly on animal-derived culture materials and co-culture with mouse-originated cells that hindered further applications of these MSCs in clinical trials. Accordingly, scientific attempts have been directed towards devising a defined culture condition to differentiate MSCs from ESCs to produce clinical-grade PSC-MSCs.

In 2007, Lian et al. attempted to generate MSCs from H1 and Hues9 hESCs using culture conditions that avoided application of any undefined components, mouse cell lines, or virus vectors used by the aforementioned methods in order to be clinically applicable [99]. In this method, hESCs were trypsinized and cultured onto a gelatin-coated culture plate using differentiation medium containing DMEM, platelet-derived growth factor AB (PDGF-AB), and bFGF for one week. Afterwards, the differentiated cells were verified for CD105^+^ and CD24^–^ using FACS sorting and gene expression analyses. CD24 represents an ESC marker. In addition, capacity of the cells to differentiate into osteocytes, adipocytes, and chondrocytes was shown.

In 2009, Karlsson et al. developed an efficient and reproducible method to generate homogenous and clinically relevant MPs from xeno-free hESCs [100] generated in a previous study [101]. This method circumvents the application of any animal-derived culture materials or factors, which were limitations of the previous trials. Accordingly, human serum for culture and recombinant human gelatin for culture dish coating substituted bovine serum and porcine-derived gelatin, respectively. In this protocol, cells were trypsinized and plated at a high cell density and cultured with differentiation medium for one week to show the emergence of heterogeneous cells. These heterogeneous cells were trypsinized and passaged for one week, and this procedure was repeated every week until obtaining homogenous cell types attained after two to three passages. These differentiated MPs did not show any surface markers of the undifferentiated ESCs and showed MSCs’ morphology with high expression of MSC-related surface markers. Upon transplantation of these cells under the kidney capsule of severely combined immunodeficient (SCID) mice for eight weeks, they efficiently differentiated into tissues of MSC origin and did not induce teratoma [100].

#### 3.1.5. MSCs Derived via Neural Crest Cells

In an attempt to avoid contamination of the finally differentiated MSCs with remnants of PSCs from the original PSCs, the intermediate cells, such as neural crest cells (NCCs), were considered as a possible source for MSC generation [102,103].

In 2014, Fukuta et al. prepared MSCs from various hESCs and hiPSCs via passing NCCs using chemically defined medium supplemented with small molecules, such as TGFβ and GSK3β inhibitors [104]. In this differentiation protocol, hPSCs were first induced to differentiate into NCCs through shifting to the induction medium composed of chemically defined medium containing the TGFβ inhibitor, SB431542, and GSK3β inhibitor, CHIR99021, for one week to allow the gradual sprawling of cranial NCCs from the colonies. The characterization of the derived cranial NCCs was based on sorting of p75^high^ cell populations. In addition, the capacity of these cells to differentiate into glia, melanocytes, peripheral neurons, and corneal epithelial cells was shown. On the other hand, a high concentration of CHIR99021 (higher than 1 µM), BMP4, SMAD1/5/8 phosphorylation inhibitor, and BMP signaling inhibitors obviously decreased the population of p75^high^ cells. The derived NCCs were maintained in chemically defined medium with SB43154, FGF2, and epidermal growth factor (EGF) for 10 passages. For MSC differentiation from the derived cranial NCCs, the maintenance medium was shifted into αMEM supplemented with 10% serum, which resulted in downregulation of NCC-related genes, such as *SOX10*, *NGFR*, *TFAP2A*, and *PAX3*; upregulation of MSC-related markers, namely, CD44, CD73, and CD105; and lack of CD45 expression. Even though the derived MSCs showed weak expression of SSEA4 and PSC-associated markers, further detailed molecular analysis of these cells needs to be scrutinized in future studies. Moreover, the in vivo application of these cells needs to be confirmed.

#### 3.1.6. MSCs Derived via the Trophoblast-Like Stage

**Figure 2:**
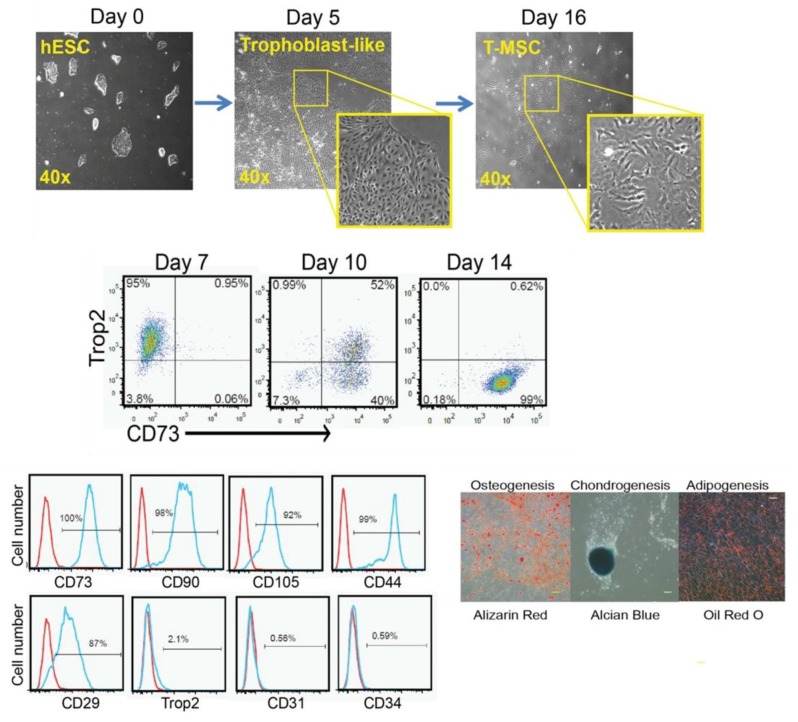
Stages of generation and characterization of human embryonic stem cells (hESC)-mesenchymal stem cells (MSCs) derived through the trophoblast-like stage as intermediate cells. The figure is reproduced from an article by Wang et al. 2016 [106] with permission from John Wiley and Sons. CD—cluster of differentiation.

The previously described classical methods for the derivation of MSCs from hESCs possess various limitations, such as the fact that they incur massive cell loss; they are laborious, time-consuming, and costly; and they generate heterogeneous MSCs, which hinder large-scale generation of MSCs for clinical applications. Trophoblasts are unique extra-embryonic cells present during early embryonic development, in particular, from the morula-to-blastocyst stage, and are involved in the formation of the chorion in the placenta. BMP4 is considered a key factor for the induction of trophoblast differentiation [105]. Wang et al. devised an innovative and efficient method for the differentiation of MSCs from hESCs using the trophoblast-like intermediate stage and in a relatively short time (about 11–16 days) (Figure 2) [106]. For trophoblast differentiation, the maintenance medium (mTeSR1 complete medium) was removed and replaced with mTeSR1 minus select factors medium, which is void of lithium chloride; bFGF; and TGFβ1 and supplemented with BMP4 and A83-01, a specific inhibitor of activin-like receptor kinases, namely ALK4, ALK5, and ALK7. The cells were maintained for five days with 5% CO_2_ at 37 °C with continuous microscopic observation to detect the dense trophoblast-like morphology. Afterwards, trophoblast-like cells were dissociated at day 5 with TrypLE and plated onto a gelatin-coated culture plate in MSC culture media, which was composed of αMEM supplemented with 20% FBS, 1% NEAA, and L-glutamine, and cultured at 37°C and 5% CO_2_ with exchange of medium every three days. The generated cells were labeled as passage 0, cultured upon reaching 80%–90% confluency, and then cultured every five to seven days in each passage. From day 11 to day 16, the formation of MSC-like cells was shown through downregulation of the trophoblast marker expression, TROP2, and the marked upregulation of CD73 (Figure 2). In particular, about 99% of the cells cultured in MSC medium differentiated to TROP2^−^/CD73^+^ cells and were thus labeled as passage 0. Detailed characterization of these cells by FACS analysis showed high expression of CD29, CD44, CD73, CD90, and CD105, whereas expression of the trophoblast, hematopoietic, and endothelial markers, such as TROP2, CD34, and CD31, was not detected (Figure 2). As these MSCs are derived through the intermediate trophoblasts, they were called trophoblast-derived MSCs (T-MSCs). The immune-modulatory function of T-MSCs was tested using anti-CD3-stimulated T cells. Similar to BM-MSC, T-MSC showed significant suppression of the proliferation of anti-CD3-stimulated T cells. Moreover, T-MSCs attenuated the proliferation of anti-IgM-activated B cells isolated from carboxyfluorescein succinimidyl ester-labeled mouse splenocytes.

T-MSCs possess low expression of MHC class-I and -II antigens, which is lower than the expression in adult MSCs. Tumor necrosis factor-alpha (TNFα)-stimulated BM-MSCs and T-MSCs showed high production of IL-6, IL-8, and CCL2. Conversely, IFNγ-activated T-MSCs did not secrete IL-6, CCL2, and CXCL10, which are highly secreted in IFNγ-activated BM-MSCs. Of note, IFNγ activation led to upregulation of the immune tolerance-inducing genes, indoleamine 2,3 dioxygenase (*IDO1*), *TGFβ1*, and *PD-L1*, in both T-MSCs and BM-MSCs, but T-MSCs sustained high expression of *PD-L1* with and without exposure to IFNγ. Further, high IFNγ did not influence the high expression level of the immune tolerance-inducing gene, *PD-L1*, in T-MSCs. Therefore, the differential immune-regulatory function between T-MSCs and adult MSCs, such BM-MSCs, is evidenced and showed the potent immune-suppressive efficacy and suitability for the in vivo transplantation of T-MSCs compared with adult MSCs. In addition, the immune-regulatory function of T-MSC was verified in vivo using the EAE model of multiple sclerosis and dextran sulfate sodium-induced colitis mouse models. Additionally, Li et al. generated MSC from hESCs using trophoblast cells as an intermediate stage, but under a complete serum free culture condition [107]. Compared with the serum-based culture condition, hESC-MSCs generated under serum free culture conditions consumed more time for differentiation but showed lower expression of the inflammatory cytokines (*IL-6* and *IL-8*) and better proliferation capacity, particularly at the late passages [107]. Taken together, using trophoblasts as an intermediate stage for the derivation of MSC from hESC is a cost-efficient and time-saving method, through which T-MSCs demonstrate efficient in vitro and in vivo immune-modulatory activity. However, the factors attributed for the potent in vivo immune-modulatory activity of T-MSC compared with other ESC-MSCs and adult MSCs need to be scrutinized in further studies.

#### 3.1.7. MSCs Derived via Spheroids Culture

ESC-MSCs can be derived using a three-dimensional (3D) platform via various methods. During the maintenance and expansion of PSCs, the suspension culture method can be performed without any changes in pluripotency or chromosomal abnormalities [108,109]. In 2017, Jiang et al. performed a comparative study on the survival rate of stem cells (hESCs, ESC-MSCs, and hBM-MSCs) cultured in a 3D culture platform (spheroids culture) or 2D platform (monolayer) under ambient conditions [110]. Stem cells cultured with a 3D system showed a higher survival rate under hypothermic conditions (from 10°C to 37°C) for a long incubation period (for 7–9 days), whereas marked cell death was detected in cells cultured in a 2D platform. In addition, the prolonged survival of hESC for four days under ambient conditions was shown.

**Figure 3:**
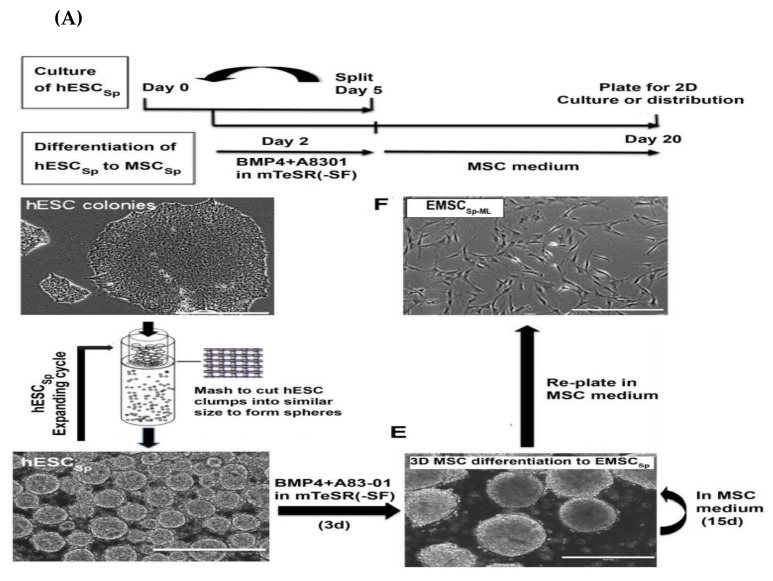

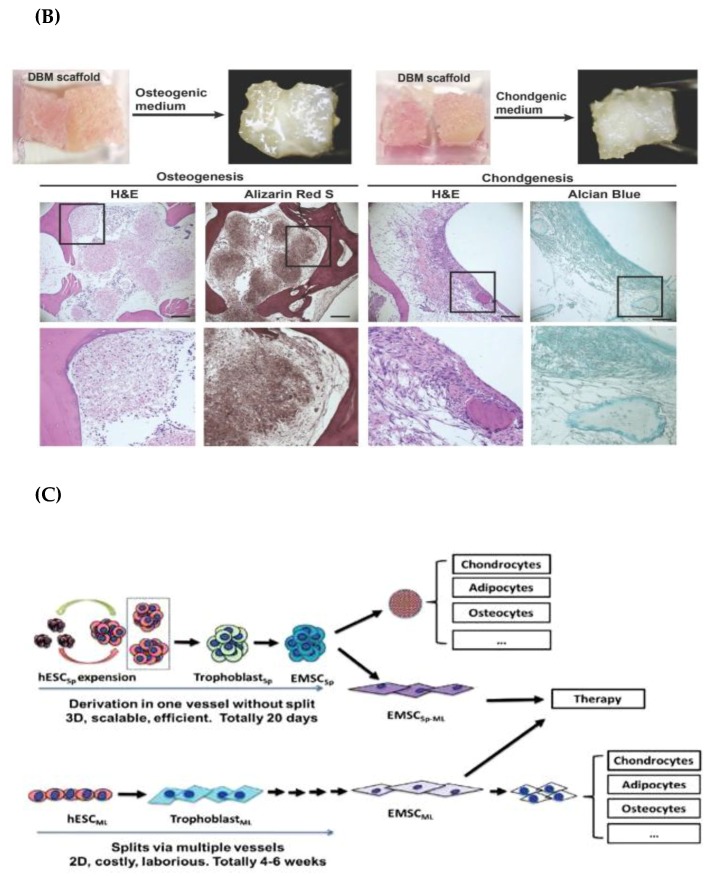
(**A**) Timetable and method for the generation of hESC-MSC_SP_ from hESC_SP_. (**B**) The osteogenic and chondrogenic differentiation of hESC-MSC_SP_ after loading in demineralized bone matrix (DBM). (**C**) Diagram summarizing the advantages of spheroid culture platform over the monolayer culture system. The figure is reproduced from the article by Yan et al. 2018 [32], which is an open access article distributed under the terms of the Creative Commons Attribution (CC BY-NC) license (https://creativecommons.org/licenses/by-nc/4.0/). BM—bone marrow; H&E—Hematoxylin and eosin stain.

Of note, re-plating of hESC spheroids (hESC_sp_) exposed to ambient conditions onto a monolayer platform showed efficient attachments and colony formation of the recovered cells when cultured under normal conditions. The higher and prolonged survival times of stem cell spheroids over the monolayer culture were ascribed to the reduced proliferation and metabolic rate [110]. On the basis of these findings, Yan et al. differentiated MSCs using a 3D platform in order to overcome shortcomings of the 2D platform-based methods for PSC-MSC derivation, such as high cost, labor, time consumption, and inefficiency [32]. In this method, homogenous hESC_sp_ were prepared according to previously reported protocols by Jiang et al. [111] and Otsuji et al. [108]. For this purpose, hESCs colonies were dissociated with dispase, washed with PBS, and then passed through a strainer (with a pore diameter of 50 μm) to ultimately produce clusters of cells of even sizes (around 50 μm) (Figure 3A). These clusters were plated on a petri dish in mTeSR1 medium supplemented with Rho-associated, coiled-coil containing protein kinase (ROCK) inhibitor, Y-27632, to prevent apoptosis. On the second day, the cultured spheroids were subjected to differentiation into trophoblast-like cells using BMP4 and A83-01, as reported by Wang et al. and described above [106]. On the third day, a significant increase in the expression level of trophoblast-related genes was detected. On the fifth day, the differentiation medium was exchanged with MSC culture medium. On day 20, the spheroids were trypsinized and transferred onto T75 flasks for further expansion in the monolayer system for further analysis of the spheroid characteristics. In accordance with the findings shown by Wang et al. [106], the positive expression of trophoblast-related markers was detected on day 5; positive expression of trophoblast- and MSC-related markers was detected on day 10; and on day 20, the positive expression of only MSC markers was shown. Moreover, this method generated around 67% more MSCs than that produced via the 2D platform. The enzymatic dissociation of cells and continual cell splitting, and plating procedures were not involved in the 3D platform; therefore, it eliminated the resultant cell loss, apoptosis, and senescence. Moreover, this method had a shorter differentiation time (20 days in 3D platform vs. 39 days in 2D platform), less technical efforts, and markedly lower culture costs compared with the monolayer method. Therefore, 3D platform-based derivation of PSC-MSCs allows for large-scale production of MSCs with faster proliferation capacity, low apoptotic changes, and low senescence for clinical applications. Interestingly, the monolayer cells that resulted from dissociation of the spheroidal hESC-MSC (hESC-MSC_SP_) showed potent immunoregulatory function evidenced in vitro via microarray assay and in vivo using a mouse model of inflammatory colitis [32]. Finally, the authors examined the potential of hESC-MSC_SP_ to adhere and differentiate in the demineralized bone matrix (DBM) scaffold, which is commonly applied in the clinical repair of cartilage and bone. Surprisingly, spheroidal hESC-MSC_SP_ markedly filled the spaces in the DBM scaffold and efficiently differentiated into bone and cartilage (Figure 3B). The authors’ conclusions showed the cost-efficient effect as well as a superior therapeutic effect of the spheroid culture over the monolayer culture platform (Figure 3C). However, these findings need to be verified using an animal model.

#### 3.1.8. MSCs Derived Using Small Molecule Inhibitors and Growth Factors

A broad spectrum of inhibitors, small molecules, and growth factors are involved in the differentiation and modulation of stem cell functions. In this line, Mahmood et al. inhibited the TGF-β/activin/nodal signaling pathway in hESCs using SB-431542 during EB formation in knockout serum replacement (KO-SR) medium for 10 days, which resulted in formation of cells characterized by high expression of paraxial mesodermal markers and myogenic-associated markers [112]. These cells were then re-plated in a fibronectin coated-culture plate in chemically defined medium supplemented with SB-431542 and insulin–transferrin–selenium, which enabled cell outgrowth and monolayer formation, and passaged up to confluency. These cells were known as SB-derived outgrowth culture and were passaged over to passage 12 without marked senescence-related changes. SB-derived outgrowth cultures were dissociated and plated in αMEM supplemented with 10% FBS for 20 days to allow derivation of the MPs that highly express CD44, CD73, CD146, and CD166, and possess the in vitro and in vivo tri-lineage differentiation capacities [112].

In 2011, Sanchez et al. enriched MSCs from hESCs via application of inhibitors targeting the SMAD-2/3 signaling pathway [113]. In this study, various hESC lines were treated with SB-431542, which is a robust chemical inhibitor that selectively blocks ALK4, ALK5, and ALK7 receptors and consequently inhibited TGF-β via targeting SMAD-2/3. After 28 days, 42% of SB-431542-exposed hESCs showed MSC-like cells that positively expressed CD73 and CD90 and were negative for CD34 expression. In addition, the tri-lineage differentiation capacity and decreased expression level of pluripotency genes, such as *Oct4* and *Tra-1-60*, were detected. Compared with umbilical cord blood-derived MSCs and BM-MSCs, hESC-MSCs showed similar proliferation capacity to umbilical cord blood-derived MSCs, but higher proliferation than that shown by BM-MSCs [113]. Of note, the efficient in vivo anti-inflammatory and immunosuppressive activities of hESC-MSCs in a mouse model of experimental colitis were shown through the increased survival rate and improved body weight loss. In 2016, Deng et al. efficiently generated MSCs from hESCs via inhibition of IκB kinase (IKK)/nuclear factor kappa B (NF-κB) signaling with a small-molecule IKK inhibitor (IKKi) [114]. In addition, IKKi maintained the normal karyotype. This study highlights the crucial role of NF-κB in the derivation of MSCs from PSCs. In addition, small molecule inhibitors are involved in the differentiation of PSCs into NCCs for the generation of MSCs, as explained above. Taken together, the small molecule inhibitors showed key roles in the derivation of MSCs from PSCs. These studies open the avenue for further research on the discovery of novel small molecule inhibitors for obtaining high-quality ESC-MSCs.

#### 3.1.9. Therapeutic Applications of ESC-MSCs in Disease Models

Verification of the therapeutic capacity of ESC-MSCs has been demonstrated in several studies. In 2012, Zhang et al. reported the therapeutic efficiency of ESC-MSCs in a monocrotaline (MCT)-induced pulmonary arterial hypertension (PAH) mouse model in comparison with that of BM-MSCs [115]. In this study, ESC-MSCs derivation protocol was according to the method of Lian et al., as described above [99]. The transplantation of ESC-MSCs via intravenous (i.v.) injection in PAH mice markedly alleviated PAH-associated thickness of the medial wall of lung arterioles, high right ventricular systolic pressure, and high degree of right ventricular hypertrophy and showed better action than that shown by BM-MSCs. Moreover, ESC-MSCs-transplanted mice showed a higher survival rate than BM-MSCs-transplanted mice. Three weeks post-transplantation, ESC-MSCs could be detected in the injured pulmonary tissue, but BM-MSCs were not detected [115]. The robust paracrine function of ESC-MSCs is implicated in their high therapeutic capacity. However, this study has weak points, such as using a mouse model rather than rat, which is less susceptible to MCT induction. Moreover, the therapeutic capacity of ESC-MSCs at the stage of PHA needs to be investigated. Taken together, this study needs to be reassessed in further investigations.

Research performed by Hawkins et al. showed that ESC-MSCs possessed a stronger neuroprotective effect in a hypoxic-ischemic mouse brain model than that shown by fetal MSCs [116]. The derivation of MSCs from ESCs was carried out using small molecules, as described previously by Chen et al. with modifications [117]. ESC-MSCs showed superior proliferation capacity than that shown by amniotic fluid-derived MSCs. In 2016, Gonzalo-Gil et al. also showed the therapeutic activity of hESC-MSCs against collagen-induced arthritis in mice [118]. The hESC-MSCs in this study were generated according to the method reported by Sánchez et al. [113]. The authors showed upregulation of regulatory T cell-mediated high IFNγ and migratory activity of hESC-MSCs to murine inguinal lymph nodes, which was accompanied by upregulation of IDO1. Therefore, hESC-MSCs are considered a powerful tool for rheumatoid arthritis therapy.

In 2015, Hajizadeh-Saffar et al. also generated hESC-MSCs based on the study by Trivedi et al. [92] and then conditionally overexpressed VEGF to finally prepare hESC-MSC/VEGF and tested their effect in the revascularization of the transplanted islet in diabetic mice [119]. Interestingly, the simultaneous transplantation of hESC-MSC/VEGF with the islet using collagen-fibrin hydrogel markedly prompted the revascularization of the transplanted islets, as well as reduced the number of transplanted islets with superior function. Taken together, this approach improved the efficacy of the transplanted islets.

**Figure 4:**
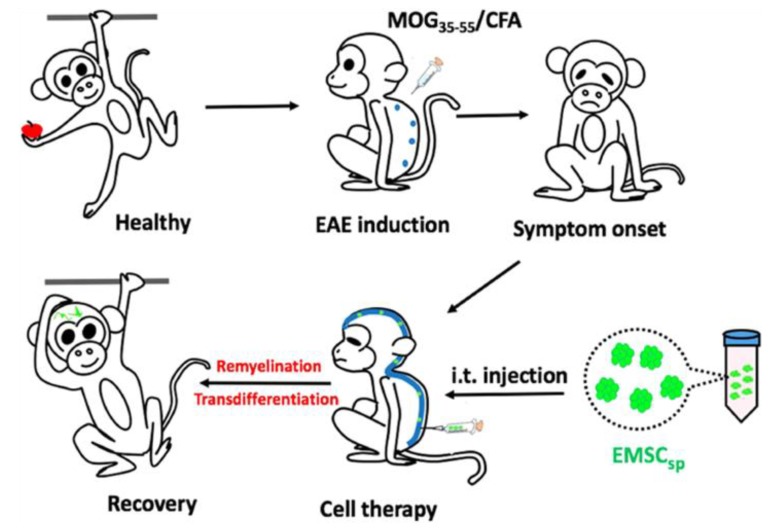
Schematic describing the therapeutic effect of hESC-MSC_SP_ in experimental autoimmune encephalitis (EAE) in monkeys. The figure is reproduced from an article by Yan et al. 2018 [120], which is an open access article distributed under the terms of the Creative Commons Attribution (CCBY-NC) license (http://creativecommons.org/licenses/by/4.0/). MOG—myelin oligodendrocyte glycoprotein; CFA—complete Freund’s adjuvant.

In 2018, Yan et al. tested the possible therapeutic efficacy of hESC-MSC_SP_ for the recovery of multiple sclerosis using the non-human primate EAE model in cynomolgus monkeys [120]. The anti-EAE capacity of hESC-MSCs was verified in the mouse model, but the anti-EAE efficacy of hESC-MSCs in non-human primate EAE needs to be evaluated to validate its feasibility in humans. There are many common immunological features between humans and non-human primates. In this study, EAE induced in cynomolgus monkeys was employed via immunization with myelin oligodendrocyte glycoprotein peptide (MOG35–55) and complete Freund’s adjuvant, and the induction was evidenced by magnetic resonance imaging (MRI) and histological assays of the central nervous system (CNS). Before application of hESC-MSC_SP_ in EAE in monkeys, they were maintained at ambient conditions for three days and one more day for transportation (total of four days in ambient conditions). Interestingly, the authors measured the viability of hESC-MSC_SP_ before injection, and it was higher than 95% [120]. The authors tested various routes for the administration of hESC-MSC_SP_, such as i.v. and intrathecal (i.t.) injections. The i.v. injection of cells led to the distribution of cells in all tissues, with the highest concentration in the CNS. However, it was eliminated within one week without further improvement of symptoms. Conversely, i.t. injection showed a direct delivery of cells into the subarachnoid cavity of the spinal cord and significantly attenuated symptoms without any noticeable side effects over a long period (for over three months) [120]. Moreover, transdifferentiation of ESC-MSCs into neural cells was detected when cultured in the cerebrospinal fluid (CSF) of EAE monkeys. In sum, hESC-MSC_SP_ without dissociation or cryopreservation efficiently diminished symptoms and progression of EAE monkeys via i.t. injection, and thus could be translated for clinical application in humans (Figure 4).

### 3.2. MSCs Derived from iPSCs

#### 3.2.1. Derivation of iPSC-MSCs via Various Culture Components and Growth Factors

iPSCs possess a high self-renewal capacity, proliferation; differentiation to mesoderm, endoderm, and ectoderm; and have bypassed ethical concerns that arise from the usage of ESCs. However, there are various challenges that hamper clinical application of iPSCs, such as immune rejection, teratoma formation, and epigenetic memory [121]. Therefore, iPSC-MSCs could be a suitable substitute for future clinical therapy, and several research groups have devised various culture media and coating materials for the production of iPSC-MSCs.

In 2010, Lian et al. created an efficient protocol for the differentiation of iPSCs to clinical-grade MSCs, which was based on using feeder-free chemically defined culture conditions and on sorting of CD105^+^ and CD24^−^ cells [122]. In this protocol, MSC differentiation was carried out using 10% KO-SR medium supplemented with a combination of growth factors, including bFGF, EGF, and PDGF-AB. Interestingly, iPSC-MSCs were identical to BM-MSCs in the expression of surface markers and tri-lineage differentiation potential. Moreover, they possessed superior proliferation capacity (up to 120 passages) without losing plasticity. Moreover, intramuscular injection (i.m.) of the differentiated iPSC-MSCs into the limb ischemia mouse model showed a therapeutic action via induction of myogenesis [122,123]. For explanation of the superior proliferative capacity of iPSC-MSCs over BM-MSCs shown by Lian et al., another mechanistic study was conducted by Zhang et al. in 2012 [124]. In this study, the electrophysiological properties of iPSC-MSCs and BM-MSCs were studied via a comprehensive profiling of the functional ionic channel, which indicated a marked increase in human ether-à-go-go 1 (hEAG1) potassium channel encoded by potassium voltage-gated channel, subfamily H (eag-related), member 1( KCNH1) [124].

In 2013, Zou et al. produced hiPSC-MSCs using a simple method based on switching of hiPSC culture medium to MSC medium with media exchanges every two days [125]. After two weeks, the cells were trypsinized and transferred onto a 0.1% gelatin-coated plate and cultured in MSC medium. After reaching confluency (3–5 days), the cells were trypsinized again. The authors obtained cells showing homogenous fibroblastic morphology after three repeats of trypsinization, and these cells were used for further characterization and differentiation. Previous reports have shown that cell passaging via repeated trypsinization can lead to enrichment of the MSC population [95,126]. The derived iPSC-MSCs highly expressed MSC-related markers and lost expression of pluripotency markers, such as OCT3/4 and TRA-1-81, but still possessed high expression of NANOG [125]. A previous study showed the crosslink between Nanog expression and the efficient proliferation and myogenic differentiation of MSCs [127]. Moreover, they successfully differentiated into osteocytes, chondrocytes, and adipocytes.

In 2014, Yang et al. generated rat iPSC-MSCs via exposure to various growth factors and under the hypoxic condition [128]. In this study, generation of rat iPSCs was carried out via reprogramming of female rat embryonic fibroblasts using lentiviral vectors. At passage 5, riPSCs were plated onto a gelatin-coated culture plate in MSC medium and then incubated in a hypoxia chamber with media exchanges every two days. Cells with fibroblastic morphology were detected after five passages of culturing with MSC medium, and these cells were similar to rat BM-MSCs, as they positively expressed CD29 and CD90, but lacked expression of CD34 and CD45.

**Figure 5:**
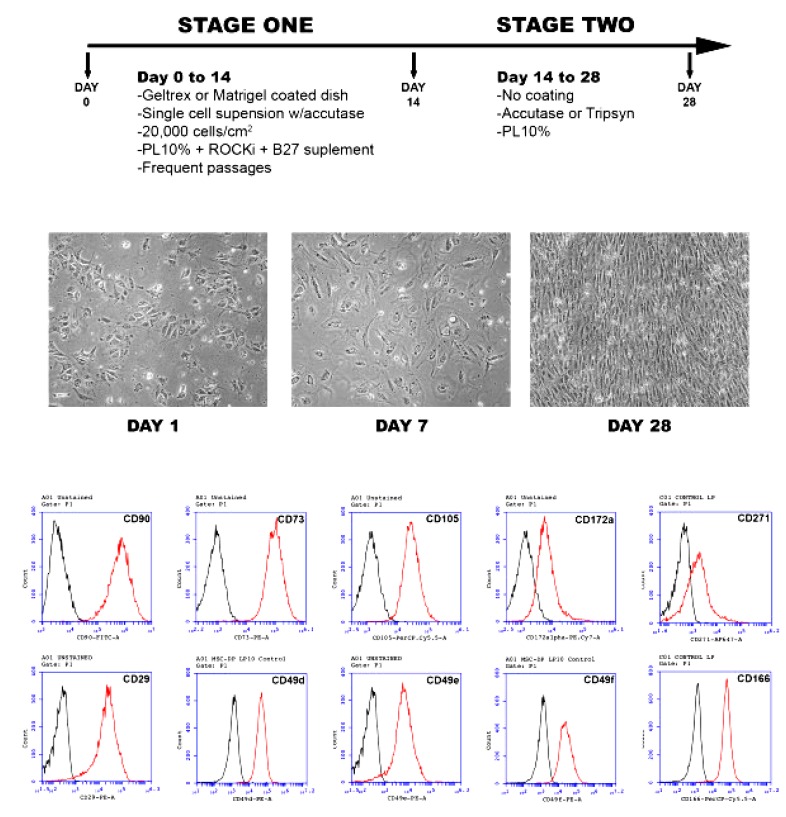
Representative figure showing the protocol for the production of induced pluripotent stem cells (iPSC)-MSCs using 10% using platelet lysate (PL) and the characterization of differentiated cells that shown in the microscopic changes in the cell morphology and the positive expression of MSC-associated markers with fluorescence-activated cell sorting (FACS) analysis. This figure is reproduced from article published by Luzzani et al. [129], which is an open access article distributed under the terms of the Creative Commons Attribution (CCBY-NC) license (http://creativecommons.org/licenses/by/4.0/).

In 2015, Luzzani et al. developed a simple and cost-effective protocol to derive MSCs from iPSCs using platelet lysate (PL) as a supplement [129]. In this protocol, iPSCs were generated by reprogramming foreskin fibroblasts and were subjected to differentiation into MSCs via exposure to αMEM supplemented with 10% PL for one month. For the first two weeks of the differentiation period, iPSCs were plated onto a Matrigel or Geltrex-coated plate with 10% PL, ROCK inhibitor, and B27 supplement (Figure 5). In the remaining two weeks, cells were maintained with only 10% PL and without coatings. At the first four days of the differentiation process, iPSC colonies showed morphological changes toward differentiation, including irregular shape, increased cytoplasmic zone, positive expression of MSC-related markers, and a significant decrease in the expression level of pluripotency-associated markers, such as Oct4 and Nanog (Figure 5).

#### 3.2.2. Derivation of iPSC-MSCs via Coating Materials and Small Molecule Inhibitors

The previous report showed the crosslink between type I collagen and the enhancement of epithelial–mesenchymal transition, which was mediated via activation of NF-κB and lymphoid enhancer-binding factor-1 (LEF-1) [130]. In this regard, Liu et al. prepared iPSC-MSCs in a one-step method, which was based on the plating of single cells of dissociated hiPSC colonies that were previously cultured in maintenance media with ROCK inhibitor, Y-27632, onto a culture plate coated with fibrillar type I collagen and maintained in culture media for 24 h [131]. Subsequently, the cells were cultured for two days in an equal volume of the maintenance medium and differentiation medium (basal αMEM supplemented with 10% FBS, dexamethasone, and magnesium L-ascorbic acid phosphate). Afterwards, the cells were cultured in the differentiation media for 10 days with exchanging media every three to four days, and the cells were harvested and cultured as passage 0 onto collagen-coated culture plates in αMEM with 10% FBS, L-glutamine, and NEAA until reaching confluence. At passage 2, the cells showed homogenous cell populations with spindle-shaped morphology, multi-lineage differentiation, and expression of MSC-associated surface markers, such as CD90, CD105, CD166, CD73, and CD146. This method exploited a collagen coating to bypass treatment with growth factors and cytokines in various steps and over a long culture time. However, the in vivo therapeutic capacity of the derived iPSC-MSCs using this one-step method needs to be verified in further studies.

In 2012, Villa Diaz et al. formulated a defined culture system for the production of iPSC-MSCs by culturing onto a poly [2-(methacryloyloxy) ethyl dimethyl-(3-sulfopropyl) ammonium hydroxide] (PMEDSAH)-coated culture plate with a defined culture medium, which bypassed the application of any animal-associated culture components [132]. PMEDSAH is a completely defined synthetic polymer coating material, which is effective in the efficient maintenance of self-renewability and the undifferentiated status of ESCs over a long-time culture [133,134]. The differentiated MSCs showed the potential for tri-lineage differentiation, expressed MSC-specific markers, such as CD44, CD73, CD105, and CD166, and did not express CD34 and CD45. Interestingly, the derived MSCs showed in vivo bone regenerative potential in a mouse model with calvaria defects [132]. However, the molecular mechanism of PMEDSAH in the derivation of iPSC-MSCs needs to be revealed in further studies. In 2012, Wei et al. also devised a one-step protocol for the simultaneous derivation of MSCs and cardiomyocytes from hiPSCs [135]. This one-step protocol is based on the formation of EBs performed using cardiac differentiation condition medium (serum- and insulin-depleted medium), which is supplemented with SB 203580 (a specific p38 mitogen-activated protein kinase (MAPK) inhibitor). Upon injection of the generated hiPSC-MSCs into immunocompromised mice, no teratoma formation was detected to confirm the safety of the generated MSCs. Moreover, the pro-angiogenic activity and wound healing property (via enhanced cellular migration) of hiPSC-MSCs were significantly higher than that shown by BM-MSCs [135]. Further in vivo validation of these derived MSCs in cardiomyopathy is needed.

In 2014, Tang et al. derived iPSC-MSCs via EB formation and then transferred them onto a gelatin-coated plate [136]. In this method, EB formation was conducted through the dissociation of iPSC colonies with collagenase type IV and plating on a low-adherent culture plate in iPSC culture medium voided of bFGF for 10 days with medium exchange every 2 days. Afterwards, the prepared EBs were plated onto a 0.1% gelatin-coated culture flask and maintained for an additional 10 days. The authors observed the adherence of EBs after two days, which was accompanied by the emergence of cells from the periphery of the EBs [136]. After reaching 70% confluency, the grown cells were picked up with scrapers and maintained in MSC culture medium. The derived iPSC-MSCs positively expressed MSC-related surface markers, whereas they lacked the expression of hematopoietic markers and pluripotency-associated markers. Interestingly, these cells showed stable adherence and good viability when seeded on calcium phosphate cement (CPC) scaffold. Moreover, iPSC-MSC-seeded CPC scaffold in osteogenic differentiation induction medium showed a 10-fold increase in ALP activity and 4-fold increase in mineralization in the osteogenic differentiation capacity compared with the control medium [136]. The in vivo validation of this model in a bone defect animal model needs to be verified in further studies.

In 2012, Chen et al. induced epithelial-like monolayer cells from iPSCs using TGF-β pathway inhibitor, SB431542, in serum-free medium [117]. After 10 days of culture, there was a marked increase in the expression level of mesoderm-related genes (*Msx2*, *Gata4*, *Runx1*, and *Bmp4*), ectodermal genes (*Cd2* and *Pax6*), and endoderm-related genes (*Sox7* and *Sox17*), which was simultaneous with the significant downregulation of pluripotency-associated genes, such as *Oct4*, *Sox2*, *Myst2*, *Lefty1*, *Lefty2*, and *Dmnt3b* [117]. Treatment of SB431542 led to suppression of SMAD2 phosphorylation and the expression of LEFTY1 and LEFTY2.

In 2013, Hynes et al. created a simple method for generation of MSCs from iPSCs derived from three various somatic tissues, namely periodontal ligament, gingiva, and lung [137]. For MSC differentiation, iPSC colonies were removed via gentle pipetting after the dissociation of MEF with collagenase type I and then transferred onto a gelatin-coated culture plate without MEF. The iPSC colonies were cultured using MSC culture media for two weeks to allow the sprawling of heterogeneous cell populations from the colonies. Afterwards, these heterogeneous cells were dissociated and plated onto gelatin-coated culture plate and labeled as passage 1. These cells were cultured onto a gelatin-coated plate for only two passages, and the authors could obtain cells with MSC morphology after 5–10 passages. FACS sorting analysis showed that more than 95% of cells expressed CD73 and CD105. In addition, they expressed the whole MSC-associated markers without expression of pluripotency markers and hematopoietic markers [137]. Of note, the authors showed the capacity of iPSC-MSCs to differentiate into osteocytes and chondrocytes is higher than differentiation into adipocytes, which requires explanation in a future study. Moreover, as the authors used various iPSCs derived from various somatic tissues, the impact of the epigenetic memory of the somatic tissue of origin on the differentiation potential of the differentiated MSCs needs to be scrutinized in further studies.

**Figure 6:**
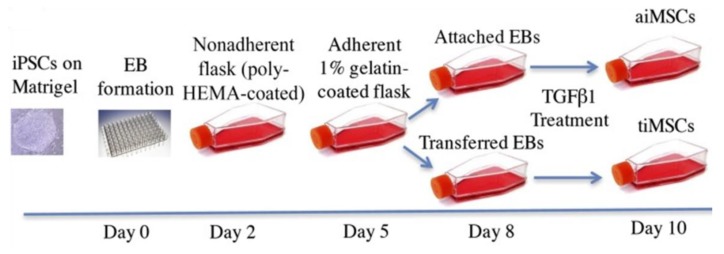
Schematic summarizing the differentiation procedure of iPSCs into 2 cell populations (attached MSCs (aiMSCs) and transferred MSCs (tiMSCs)). This diagram is reproduced from articles by Sheyn et al. [138] following permission from John Wiley and Sons. TGF-β1—transforming growth factor-beta 1. EB—embryoid bodies; HEMA—hydroxyethyl methacrylate.

In 2016, Sheyn et al. differentiated iPSCs to MSCs through treating EBs with transforming growth factor-beta 1 (TGF-β1) for a short period [138]. In this protocol, for EB formation, iPSCs were dissociated using Versene EDTA and then plated onto non-adherent polymerase chain reaction plates in Iscove’s modified Dulbecco’s medium (IMDM) (MDM basal media, 17% KO-SR, 1% MEM-NEAA, and 1% antibiotic-antimycotic solution) (Figure 6). On the second day, the formed EBs were moved to non-adherent poly-hydroxyethyl methacrylate-coated flasks for three days. On the fifth day, EBs were moved again onto 1% gelatin-coated flasks and cultured until day 8. Afterwards, the authors observed the adherence of some EBs to the flask surface and sprawling of cells from EBs, and the non-adhered EBs were transferred onto gelatin-coated flasks again. On the basis of this process, the authors classified the derived cells into two groups, including attached cells (aiMSCs) that derived from EBs between day 2 and day 5 (early stage) and the transferred cells (tiMSCs), which were obtained from EBs that transferred into another gelatin-coated flask between day 5 and day 8 (late stage) (Figure 6). From day 8 to day 10, the attached and transferred cells were cultured in standard DMEM culture medium containing 10% FBS, and L-glutamine supplemented with TGF-β1. Both aiMSCs and tiMSCs shared a similar expression level of CD44, CD90, and CD105 compared with BM-MSCs, but showed a markedly higher proliferation rate than that shown by BM-MSCs. At passage 5, the highest doubling rate was detected for iMSCs (around 1.8 doublings/ day) compared with BM-MSCs, which showed around 1.8 doublings/day. Of note, aiMSCs showed a significantly higher cell doubling rate at passage 3 than the doubling rate of BM-MSCs. Interestingly, the significant upregulated expression of osteogenic differentiation markers, such as ALP and collagen type1 at the early stage of differentiation (after one week) was detected in tiMSCs, but not in aiMSCs or BM-MSCs. iMSCs showed lower tumorigenicity than that shown by BM-MSCs, which validated by colony-forming potential using the soft agar. The tri-lineage differentiation was shown in both iMSCs, but the osteogenic differentiation capacity was obviously higher in aiMSCs than in tiMSCs or BM-MSCs [138]. The molecular mechanism involved in the high osteogenic differentiation of aiMSCs needs to be revealed in further studies.

#### 3.2.3. Derivation of iPSC-MSCs via Ectopic Expression of MSC-Related Genes

In addition to the previously described methods, MSCs can be derived from iPSCs that ectopically overexpress MSC-related genes or transcription factors. In this regard, Steens et al. generated vascular wall (VW)-derived MSCs from miPSC that overexpress VW-derived MSC-associated *HOX* genes [139]. In this study, miPSCs were generated from the mouse tail-tip-derived fibroblast via transduction of the Yamanaka factors using lentiviral vectors. This tail-tip-derived fibroblast was originated from a transgenic mouse, in which the green fluorescent protein (*GFP*) gene was expressed via regulation of the Nestin promoter, to easily track the differentiation of miPSCs to MSCs. Therefore, the generated miPSC was collectively called NEST-GFP iPSC. Further, the NEST-GFP iPSC was transduced with a self-inactivating lentiviral vector that co-expressed VW-derived MSC-associated *HOX* genes, namely *HOXB7*, *HOXC6*, and *HOXC8*. MSCs’ differentiation was performed via EBs’ formation and then incubation in MSC media, complete DMEM supplemented with 20% serum. The authors confirmed the high ectopic expression of HOX proteins in the differentiated EBs with Western blotting and FACS analyses, which was simultaneous with the expression of *GFP* and *Nestin* [139]. *HOX*-expressing EBs showed efficient differentiation to the multipotent *GFP*-expressing MSCs. In sum, overexpression of *HOX* genes markedly promoted iPSC differentiation into MSCs that positively expressed *GFP* and *Nestin* and was associated with vessels in the in vivo teratoma. This study affords a potent model that opens the door for ectopic expression of favorable MSC-related therapeutic genes. However, the feasibility of this model needs to be verified in human cells.

#### 3.2.4. Derivation of iPSC-MSCs via NCCs

As we described above, NCCs could be an intermediate stage for the derivation of MSCs from ESCs. NCCs can be also used for the production of iPSC-MSCs. In 2016, Ouchi et al. induced NC-like cells (NCLCs) differentiation from hiPSCs, and they found that hiPSC-derived NCCs highly expressing low-affinity nerve growth factor receptor (LNGFR) and thymocyte antigen-1 (THY-1) (LNGFR^+^ THY-1^+^ NCLCs) potentially differentiated into MSCs [140]. In this protocol, hPSCs were cultured in neural crest medium, as described by Bajpai et al. [141], for one day. Afterwards, removal of dead cells was carried out by exchanging medium. On the fourth day, only spheroids were picked and then transferred onto an adherent culture plate. Between day 9 and day 10, the emergence of spindle-shaped NCCs from the spheroids was observed. FACS analysis showed high expression of LNGFR and THY-1 in these cells. LNGFR and THY-1 were considered specific surface markers for MSCs [142]. In addition, LNGFR is widely expressed in MSCs [73,143], but not in hematopoietic cells [73]. LNGFR^+^ THY-1^+^ NCLCs showed active mobility and can differentiate into osteocytes, adipocytes, chondrocytes, and neural crest-related cells [140]. Moreover, upon transplantation of LNGFR^+^ THY-1^+^ NCLCs into chicken embryos, the cells showed proliferative capacity and could survive in the in vivo environment, migrating to the sclerotome region. However, the in vivo differentiation potential was limited. Taken together, NCCs could be suitable intermediate cells for the derivation of multipotent MSCs from iPSCs.

In 2018, Kimura et al. examined the in vivo application of iPSC-derived LNGFR^+^ THY-1^+^ NCLCs in the regeneration of peripheral nerve defects [144]. In this study, NOD/SCID mice with sciatic nerve injury were used for evaluating the therapeutic effect of iPSC-derived LNGFR^+^ THY-1^+^ NCLCs in the recovery of nerve injury. For cell transplantation, cells were first mixed with type I collagen and then filled in silicone tubes. Interestingly, the transplanted cells significantly enhanced axonal growth, angiogenesis, and remyelination. They also recovered the disturbed motor function of the defected nerves, suggesting that iPSC-derived LNGFR^+^ THY-1^+^ NCLCs are a promising tool for therapy of peripheral nerve injuries in the future.

#### 3.2.5. iPSC-MSCs with Immunomodulatory and Anti-Inflammatory Functions

In 2011, Giuliani et al. produced iPSC-MSCs possessing an immunomodulatory function [145]. The differentiation of MSCs from iPSC and hESCs was carried out using DMEM/F12 medium containing 10% FBS, b-FGF, L-glutamine, and ß-mercaptoethanol. After one month, the authors detected spindle-shaped adherent cells, which expressed MSC-related markers and did not express hematopoietic genes. The expression of reprogramming factors (*Oct3/4*, *Sox2*, *Nanog*, and *Lin28*) was completely abrogated after differentiation. The iPSC-MSCs shared common characteristics, such as morphology and immunomodulatory functions, with the parental hESC-MSC. In this regard, both iPSC-MSCs and hESC-MSCs obviously suppressed the proliferation of natural killer (NK) cells and abolished its cytolytic activity. This potent immunomodulatory action is attributed to the suppression of the ERK1/2 signaling pathway, which hampered the formation of the immunologic synapse with target cells and the consequent production of secretory granules. Of note, iPSC-MSCs and hESC-MSCs showed a stronger resistance against the killing of pre-activated NK cells than that shown by BM-MSCs. Interestingly, iPSC-MSCs maintained the suppressive action against the proliferation of NK cells, even after 10 passages, in contrast to BM-MSCs, which showed a decline in its immunosuppressive function after three passages [145]. Therefore, iPSC-MSCs could be a powerful and long-lasting alternative cell source for immunomodulatory adult MSCs and could eliminate allograft rejection.

Another interesting research study was carried out by Frobel et al., in which MSCs were redifferentiated from iPSC derived from the reprogramming of BM-MSCs, and the resultant MSCs were called iPSC-MSCs [146]. BM-MSC-derived iPSCs used in this study were established according to the method reported by Shao et al. in 2013 [147]. The redifferentiation of this iPSCs to iPSC-MSCs was performed via culture using MSC standard medium supplemented with 10% human PL for one week or EB formation for one week and then passaging in a coated or non-coated plate. iPSC-MSCs shared similarities with the original MSCs, including morphology, gene expression profiles, tri-lineage differentiation potential, and immunophenotypic properties. In contrast, the iPSC-MSCs showed markedly lower immunomodulatory function with lower expression of T cell activation- and immune response-related genes than that detected in the original MSCs. Of note, the authors analyzed the epigenetic alteration between the original MSCs and iPSC-MSCs through DNA methylation profiles that showed abrogation of age-associated and tissue-specific DNA methylation patterns in the iPSC-MSCs compared with the original MSCs. Therefore, there is a clear difference between the iPSC-MSCs and original MSCs in the immuomodulation and DNA methylation profiles [146]. The weak immunomodulation function of iPSC-MSCs, especially their diminished function to suppress T cell activation, hampers further clinical application of iPSC-MSCs. This immunomodulation variation between iPSC-MSCs and their original MSCs needs to be investigated in further research work.

In 2015, Zhao et al. devised a modified method for generation of iPSC-MSC using a SMAD-2/3 inhibitor (SB-431542) [148]. After approximately 25 days, spontaneous differentiation was detected, which was characterized by the emergence of large spindle-shaped cells with a large cytoplasmic space. Afterwards, these cells were trypsinized into single cells and plated onto a standard cell culture plate in medium for ESC-MSCs [126] to obtain a large number of adherent cells with spindle-shaped fibroblasts. Significant downregulation of the pluripotency and neuroectodermal markers, whereas obvious increases in the expression level of mesodermal markers, were detected after about 45 days. Moreover, the derived iPSC-MSCs possessed similar characteristics to that of BM-MSCs [148]. In this report, iPSC-MSCs showed a similar tumor-homing property to BM-MSCs, but did not enhance cancer stemness, pro-EMT, and cancer invasion, as shown by BM-MSCs [148]. These iPSC-MSCs with low pro-tumor activity could be promising cells for anti-cancer activity and for the safe delivery of anti-cancer drugs compared with BM-MSCs, which promoted cancer progression and invasiveness.

In 2015, Sun et al. characterized the immune privilege property of the purchased of commercially produced iPSC-MSCs derived from fetal and adult BM via estimation of the expression level of human leukocyte antigen (HLA) after exposure to interferon-γ (IFN-γ) in comparison with BM-MSC and fetal MSCs [149]. After two and seven days of IFN-γ exposure, BM-MSCs and fetal MSCs showed a marked increase in the expression level of HLA-II, whereas iPSC-MSCs did not express HLA-II. However, the expression level of HLA-II was much higher in BM-MSCs than in fetal MSCs. In contrast, HLA-I was expressed in all the tested MSCs, and there was no significant difference in its expression among the tested MSCs. In addition, the expression of HLA-II related signaling and genes, including the phosphorylation of signal transducer and activator of transcription 1 (STAT1) signaling and expression of Interferon Regulatory Factor 1 (*IRF-1*) and class II major histocompatibility complex transactivator *(CIITA)* genes, showed a similar expression pattern to that in the expression of HLA-II among the tested MSCs after IFN-γ stimulation [149]. For in vivo confirmation of the in vitro findings, an immune humanized NOD/SCID gamma mouse with induced hind limb ischemia was transplanted with BM-MSCs and iPSC-MSCs. More iPSC-MSCs were retained in the ischemic limb than BM-MSCs, and the marked attenuation of inflammatory changes in iPSC-MSC-transplanted mice was detected compared with the BM-MSC-transplanted group. Of note, BM-MSC-transplanted mice showed high expression of HLA-II, whereas no expression was detected in the iPSC-MSC-transplanted group [149]. In sum, this study was the first study to analyze the immune privilege of iPSC-MSCs in vivo and in vitro and showed the robust immune privilege compared to the adult MSCs, suggesting that iPSC-MSCs could be a potent alternative to adult MSCs in allogeneic transplantation.

In 2017, Gao et al. showed the impact of iPSC-MSC on the molecular functions of the dendritic cells (DCs), such as maturation and differentiation [150]. In this study, MSCs were generated from urine cell-derived-iPSC using the method by Lian et al. [122]. Of note, the iPSCs were generated via electroporation without viral-based vectors or c-MYC and cultured in xenogeneic-free culture conditions, as described by Xue et al. and Wang et al. [151,152]. The derived iPSC-MSCs showed similar characteristics of BM-MSCs and showed a superior proliferation capacity to that shown by BM-MSC. iPSC-MSCs shared a common immunoregulatory function as shown by BM-MSCs, but the immunoregulatory function of iPSC-MSC was sustained even after the advanced passages (passage 18), whereas it markedly declined in BM-MSCs after passage 8. Moreover, iPSC-MSCs had a suppressive action on the differentiation of CD14^+^ monocytes to DCs, particularly at the early stage of differentiation. Interestingly, iPSC-MSCs boosted phagocytic capacity and abrogated T-cell activation potential of DCs. Moreover, during DC maturation, iPSC-MSCs promoted the formation of regulatory DCs that produced IL-10, and this attributed to cell–cell interaction mechanisms [150]. Taken together, this study evidenced the efficacy of iPSC-MSC in the modulation of DC differentiation and immunogenic properties and, accordingly, could be a potent therapeutic tool against DC-related allergic diseases. However, this model needs in vivo verification in future studies.

In 2018, Wang et al. estimated the effect of glucocorticoids, such as dexamethasone, on the immunomodulatory function of iPSC-MSCs in vitro and in vivo [153]. For this purpose, the authors used anti-CD3/CD28-stimulated peripheral blood mononuclear cells (PBMCs) and a mouse model of allergic airway inflammation and contact hypersensitivity for the in vitro and in vivo evaluations, respectively. In this study, the differentiation of iPSC-MSCs was carried out with a modified protocol, which was based on previous methods by Giuliani et al. [145] and Hynes et al. [137]. The authors did not detect any effect of dexamethasone on the immunosuppressive function of iPSC-MSCs on the proliferation of anti-CD3/CD28-stimulated PBMCs. Similarly, dexamethasone did not antagonize the immunomodulatory function of iPSC-MSCs on the allergic airway inflammation and local inflammation in the regional lymph node in contact hypersensitivity in mice. However, the treatment of dexamethasone alone showed a stronger immunomodulatory function on the ear thickness of contact hypersensitivity mice than that shown by iPSC-MSCs or iPSC-MSCs co-treated with dexamethasone [153]. In contrast, a previous study showed the antagonistic effect of dexamethasone on the immunoinhibitory effect of BM-MSCs on the proliferation of anti-CD3-induced T-cells [154]. Accordingly, iPSC-MSCs are safer to be applied simultaneously with steroids in clinical settings than adult MSCs.

The anti-inflammatory activity of riPSC-MSCs generated by Yang et al. was shown [128] and, in the study, the authors overexpressed the tumor necrosis factor alpha-stimulated gene-6 (*TSG-6*) in the derived riPSC-MSCs (riPSC-MSCs/TSG-6) using lentiviral vectors. Further, they examined their in vivo anti-inflammatory activity using a rat model of experimental periodontitis via inoculation of *Porphyromonas gingivalis* (*P. gingivalis*). The i.v. injection as well as topical application (cells encapsulated in the Matrigel) of riPSC-MSCs and riPSC-MSCs/*TSG-6* was performed once weekly for three weeks. The systemic administration of riPSC-MSCs/*TSG-6* significantly alleviated the inflammatory events shown by the reduction in the concentrations of the pro-inflammatory cytokines, namely TNF-α and IL-1β, in the serum of riPSC-MSCs/*TSG-6*-injected rats. Moreover, the riPSC-MSCs/*TSG-6*-treated group showed a significant rescue of alveolar bone loss and blocked the osteoclast formation implicated in bone resorption [128]. Taken together, this study affords a model of therapeutic application of riPSC-MSCs/*TSG-6* for dental diseases. However, the application of this model using human cells needs to be investigated.

#### 3.2.6. iPSC-MSCs for Bone Regeneration

In 2014, a comparative study was carried out to generate MSCs from iPSCs generated through reprogramming of BM-MSC with lentiviral-expression of pluripotency factors, *Oct3/4*, *Sox2*, *Klf4* and *c-Myc* [155]. In this study, generation of iPSC-MSCs was carried out using the previously described methods, such as EB formation [156], spontaneous differentiation [92], BM-MSC growth medium [157], and indirect co-culture [158]. Subsequently, the authors compared the characteristics of iPSC-MSCs with the original or parental BM-MSCs. iPSC-MSCs shared the same morphology and expression level of MSC-associated surface markers as the parental BM-MSCs. In contrast, the tri-lineage differentiation capacity, particularly the adipogenic differentiation and expression level of pluripotency genes in iPSC-MSCs, was markedly lower than that in the parental BM-MSCs. These findings conclude that iPSC-MSCs and the parental BM-MSCs are not completely identical. Of note, the weak adipogenic differentiation capacity of PSC-MSCs was also shown in previous reports [117,146,159], which needs further explanation. Further detailed studies that explain the molecular factors implicated in the differences between PSC-MSCs and parental MSCs are necessary. Moreover, the therapeutic efficacy of aiMSCs and tiMSCs developed by Sheyn et al. for bone regeneration was examined in comparison with BM-MSCs [138]. For that, the transfection of the osteogenic gene, *BMP6*, with non-viral vectors was carried out using the nucleofection. Interestingly, the secretion of the BMP6 protein into the media of tiMSC is markedly higher than that detected in the media of BM-MSCs and aiMSCs. One day after the nucleofection, the BMP6-nucleofected iMSCs and BM-MSCs were injected into the thigh muscle of NOD/SCID mice for the further examination of the in vivo ectopic bone formation. One-month post-injection, quantitative µCT analysis showed a similar pattern of high ectopic bone formation after BM-MSCs or aiMSCs injection, whereas injection of tiMSCs showed a weak ectopic bone formation. In addition, the capacity of BMP6-overexpressing iMSCs and BM-MSCs to regenerate nonunion radial defects in immune-compromised mice model was monitored. Four and eight weeks after the surgery, treatment of tiMSCs led to obvious regeneration of the bone defects via generating a higher volume of bone in the defect region than BM-MSCs and aiMSCs [138]. The significant effect of tiMSCs in regenerating the radial defects over BM-MSCs or aiMSCs is attributed to their higher doubling rate at the early passage, higher expression level of the osteogenic genes at the early stage, and high production of BMP6 in the medium compared with the other tested MSCs. However, the effect of iMSCs in other bone defect models needs to be verified in further studies.

iPSC-MSCs differentiated in the study by Zou et al. showed high functional compatibility with the 3D synthetic polymer polycaprolactone (PCL) scaffolds, which were functionalized with the natural polymer hyaluronan and ceramic TCP (PHT) [125]. iPSC-MSCs in the scaffolds showed a markedly high in vitro osteogenic differentiation capacity, which was shown in high accumulation of calcium and upregulation of ALP. The s.c. implantation of iPSC-MSCs seeded in the scaffold in the nude mice showed high mineralization, but this was not shown upon implantation of the scaffold only. These data showed the efficacy of iPSC-MSCs in the scaffold to enhance ectopic bone formation. These findings demonstrate the compatibility of iPSC-MSCs to be seeded in scaffolds for orthopedic applications.

#### 3.2.7. iPSC-MSCs for Diabetes Therapy

The in vivo anti-diabetic effect of iPSC-MSCs was also reported in the previous studies. In 2013, Himeno et al. showed the anti-diabetic function of miPSC-MSCs [160]. miPSC-MSCs were derived by firstly preparing EBs for two days, followed by culturing of EBs for three days in differentiation medium supplemented with all trans retinoic acid. Next, the repeated passage of the colonies via the enzymatic dissociation was carried out in culture plate for four months. The transplantation of miPSC-MSCs via the i.m. injection of streptozotocin-diabetic mice led to a noticeable alleviation of diabetic polyneuropathy. The authors showed homing of the injected cells in the hindlimb muscles and peripheral nerves. In the peripheral nerve site, the transplanted MSCs expressed S100***β***, a Schwann cell marker, which denotes their differentiation into the peripheral nerves. Therefore, iPSC-MSCs showed a high capacity for the attenuation of the diabetic-related neuropathy.

In 2015, Cheng et al. showed that the combination of iPSC-MSCs with a low concentration of rapamycin resulted in prolongation of the survival rate of the islet allograft in the diabetic mouse model [161]. Compared with rapamycin alone, iPSC-MSCs combined with rapamycin showed downregulation of CD4^+^ and CD8^+^ T cells, increase in the T regulatory cells, downregulation of interferon-γ and IL-2, and high production of IL-10 and TGF-β. This combination avoids the toxic action of the high concentration of the rapamycin.

#### 3.2.8. Other Therapeutic Efficacies of iPSC-MSCs

In 2015, Zhang et al. characterized the potency of the paracrine function of hiPSC-MSCs against doxorubicin (DOX)-mediated cardiomyopathy in comparison with BM-MSCs [162]. Generation of hiPSC-MSCs in this study is performed according to the protocol by Lian et al. [122]. For the evaluation of the protective action against DOX-mediated cardiomyopathy, the authors used a CM of hiPSC-MSC and BM-MSCs; the preparation of the CM was performed as described by Sze et al. [163]. The CM of hiPSC-MSCs showed a better effect in the suppression of reactive oxygen species (ROS) generation and alleviation of the apoptosis in DOX-mediated cardiomyopathy than that shown in CM of BM-MSCs. Cytokines assay showed the enrichment of hiPSC-MSC-CM with a wide-range of factors with anti-inflammatory, anti-oxidant, and anti-apoptotic functions, in particular, a high level of the cytokines growth/differentiation factor (GDF)-15 and macrophage inhibitory factor (MIF). Of note, the expression of these cytokines in hiPSC-MSC-CM was markedly higher than the CM of BM-MSCs. The depletion of MIF and GDF-15 with the immunoprecipitation from the hiPSC-MSC-CM led to obvious abrogation of the anti-oxidant and the anti-apoptotic functions, respectively. The confirmation of the in vitro activity of the CM was carried out using a primary culture of the neonatal rat cardiomyocytes that isolated from neonatal Wistar rats and these cells were subjected to DOX treatment. The in vitro data were verified using an in vivo mouse model, in which the cardiomyopathy was induced via the intraperitoneal injection of DOX. The injection of the tested CM was employed via the direct intramyocardial injection with a single dose. Consistent with the in vitro findings, hiPSC-MSC-CM alleviated DOX-mediated cardiomyopathy in mice. Moreover, injection of the CM that depleted MIF and GDF-15 showed a drastic decline in the therapeutic effect against DOX-mediated cardiomyopathy in mice. Collectively, MIF and GDF-15 are the key mediators involved in the potent paracrine-mediated therapeutic efficacy of hiPSC-MSC-CM against DOX-induced cardiomyopathy in vitro and in vivo. However, the upregulated factors in the cytokine assay other than MIF and GDF-15 need to be investigated in further studies.

For the comparison between iPSC-MSCs and MSCs in the immunomodulation, Fu et al. examined the potency of iPSC-MSCs and BM-MSCs to modulate the proliferation of lymphocytes and the response of the regulatory T-cell after the co-culture of both cells with the PBMCs of patients suffering from allergic rhinitis [164]. In this study, iPSC-MSCs were generated as described earlier by Lian et al. [122]. The phenotype for immunosuppression of iPSC-MSCs was similar to that of BM-MSCs [164]. As shown by BM-MSCs, iPSC-MSCs significantly suppressed lymphocytes proliferation induced by phytohaemagglutinin and the cytokine profiles in the supernatant of the PBMCs. Multi-color FACS analysis showed the accumulation of the regulatory T cells in G3 and G4 phases upon exposure to iPSC-MSCs or BM-MSCs. Moreover, the marked decrease in the proliferation of T cells positive for CD3 was detected. Production of prostaglandin E2 and cell contact was implicated in the immunomodulatory function of iPSC-MSCs or BM-MSCs. Interestingly, the immunosuppressive capacity of iPSC-MSCs was sustained even after various passages (10 passages) but declined after three passages for BM-MSCs. Taken together, this study affords iPSC-MSCs as a powerful and promising tool for the treatment of the allergic inflammation. However, the in vitro findings of this study need further verification using an in vivo inflammation mouse model.

In 2018, Hynes et al. derived MSCs from miPSCs, which were generated from the reprogramming of tail-tip fibroblasts isolated from NOD/Lt mice [165]. The differentiation of MSCs from miPSCs was carried out by the methods devised by the same research group [137,166]. Using FACS analysis, miPSC-MSCs showed high expression of CD73, CD105, and Sca-1 and were deficient in the expression levels of CD34, CD45, and SSEA1. The in vitro and in vivo immunomodulatory activity of miPSC-MSCs was investigated using concanavalin A-activated mouse splenocyte and periodontitis sponge mouse model, respectively. Co-culture of miPSC-MSCs with concanavalin A-activated mouse splenocytes led to marked suppression of concanavalin A-induced proliferation. In addition, the s.c. injection of miPSC-MSCs significantly suppressed the inflammation in the mouse model implanted with *P. gingivalis* containing sponge [165]. Taken together, MSCs derived from miPSCs showed a potent anti-inflammatory action that could be a promising tool for inflammatory diseases therapy, such as rheumatoid.

The efficacy of iPSC-MSCs generated in a study by Lian et al. [122] alleviated the cigarette smoke-induced damage in a rat model [167]. The i.v. administration of iPSC-MSCs in cigarette smoke-exposed rats showed a significantly higher capacity in rescuing the severe damage of the alveolar epithelium than that shown by the administered BM-MSCs. The potent action of iPSC-MSCs is attributed to the transferred mitochondria from iPSC-MSCs to the damaged airway epithelium and their role in alleviating the damage. The in vitro mitochondrial transfer was examined using the bronchial epithelial cells (BEAS-2B) and the mitochondrial transfer from iPSC-MSCs or BM-MSCs was carried out through the tunneling nanotubes (a sensitive nanotubular structure made between the cells). The rate of the mitochondrial transfer from iPSC-MSCs to BEAS-2B cells was significantly higher than the rate shown by BM-MSCs. In particular, the co-culture of iPSC-MSCs with BEAS-2B cells exposed to 2% of cigarette smoke medium showed a higher mitochondrial transfer rate than that with BM-MSCs and confirms the role of cigarette smoke medium for promoting the mitochondrial transfer from iPSC-MSCs. Of note, the co-culture with iPSC-MSCs potently rescued the cigarette smoke medium-mediated drastic decrease of the level of the intracellular adenosine triphosphate (ATP), whereas co-culture with BM-MSCs showed a weak action. Taken together, iPSC-MSCs showed a superior effect in the therapy of the pulmonary disorders over the adult MSCs. Additionally, iPSC-MSCs can be generated from patients with various diseases for studying disease pathogenicity and for the further discovery of novel drugs. This subject is summarized and discussed elsewhere [168].

**Figure 7:**
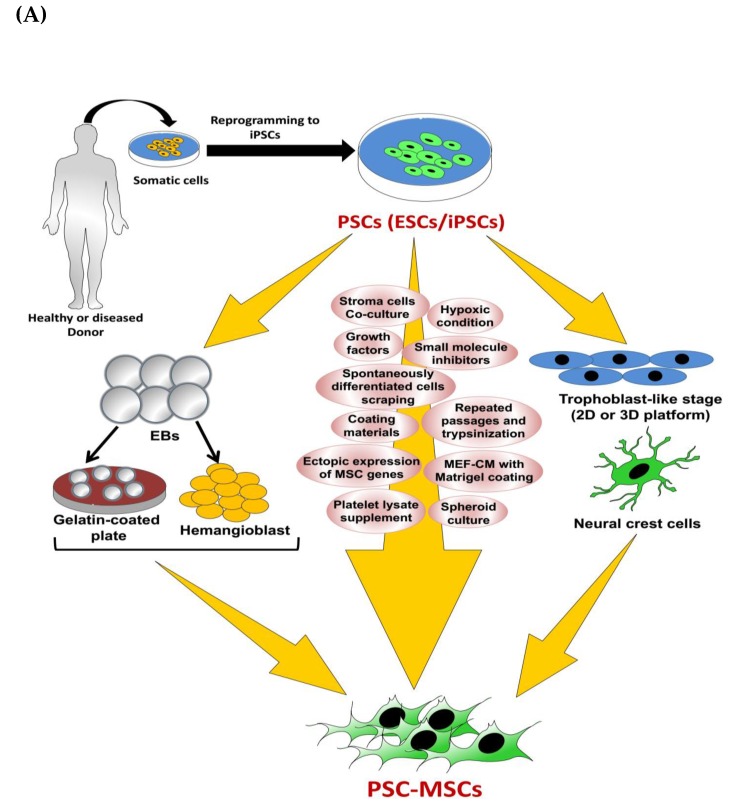

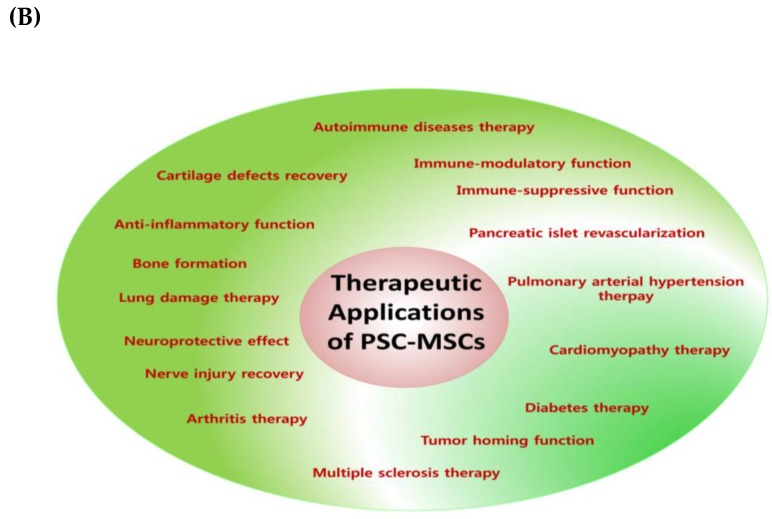
Schematic diagram outlining the methods for pluripotent stem cells-derived mesenchymal stem cells (PSC-MSCs) production and their therapeutic applications. (**A**) Methods for the production of PSC-MSCs. (**B**) The therapeutic applications of PSC-MSCs in various diseases. EBs—embryonic bodies; MEF—mouse embryonic fibroblast; CM—conditioned medium; ESC—embryonic stem cells.

The exosomes isolated from iPSC-MSCs play important roles in the therapeutic applications of iPSC-MSCs. For instance, the exosomes isolated from iPSC-MSCs (iPSC-MSC-Exo) showed a superior therapeutic activity in the attenuation of the experimental collagenase-induced osteoarthritis (OA) mouse model than that shown by the exosomes purified from MSCs derived from the synovial membrane (SM-MSC-Exo) [169]. Additionally, iPSC-MSC-Exo significantly promoted the proliferation as well as the migration of the chondrocytes, and these effects were higher than that induced by SM-MSC-Exo. In 2015, Hu et al. showed the capacity of iPSC-MSC-Exo in the alleviation of mouse ischemic limb injury via promoting the density of the microvessels and the perfusion of the blood [170]. This potent activity is attributed to the iPSC-MSC-Exo-related induction of angiogenesis. In 2015, Zhang et al. showed the role of iPSC-MSC-Exo in the healing of the cutaneous wound shown in promoting the re-epithelialization, enhanced the collagen maturity, and decreasing scar width [171]. Activation of the angiogenesis and promotion of the collagen synthesis were also implicated in iPSC-MSC-Exo-mediated wound healing [171]. The methods of derivation and the therapeutic efficacies of PSC-MSCs are summarized in Table 1. Additionally, we outline the methods for production of PSC-MSCs and their therapeutic applications in Figure 7.

## 4. Two-Edged Sword: Properties of PSC-MSCs and Future Prospects

Recently, various clinical trials have examined the therapeutic application of MSCs against a wide range of diseases, namely arthritic diseases, autoimmune diseases, and wound healing. The immunoprivileges of MSCs, such as expression of HLA-G and MHC class I molecules (non-canonical), the lack of expression of co-stimulatory molecules (CD40 and CD80), and expression of the serine protease inhibitor that escapes the immune reaction, have been implicated in their therapeutic applications [172,173]. MSCs can be isolated from various tissue in a high amount. However, there is an urgent need to generate MSCs of high quality instead of quantity in order to be efficiently applied in clinics. PSCs could be an important source to produce high-quality MSCs with potent therapeutic capacities.

Above, we described the various aspects of methodology, characterization, and therapeutic applications of PSC-MSC derivation in comparison with those of primary MSCs. Most research studies that have analyzed PSC-MSC characteristics showed their similarity with tissue-derived MSCs and possible application for regenerative medicine [89,99]. MSCs obtained from various tissue sources possessed similar molecular characteristics, immunological features, paracrine action, proliferation, and differentiation capacities, but the factors implicated in these similarity and variations need to be further scrutinized [174,175].

Even though PSC-MSCs and various MSCs derived from different tissue sources share similar morphology, surface antigen expression levels, immunophenotypes, and tri-lineage differentiation capacity, there are differences in their therapeutic capacities and their gene and protein profiles. In this regard, BM-MSCs and AD-MSCs showed a marked increase in the expression level of cytoskeletal proteins [174]. In contrast, placenta-derived MSCs showed increases in the expression level of apoptotic proteins, oxidative stress-related proteins, and peroxiredoxin proteins [174]. Accordingly, placenta-derived MSCs showed higher therapeutic capacity against the hindlimb ischemic disease than other types of MSCs [174]. Moreover, AD-MSCs potently suppressed the activation of T, B, and natural killer (NK) cells and inhibited T cell activation at the early stage, which was significantly stronger than the activity shown by BM-MSCs and umbilical cord matrix-derived MSCs [176]. Moreover, the therapeutic efficacy of BM-MSCs is relatively lower than other MSCs derived from different sources [176,177,178,179]. Collectively, application of BM-MSCs as standard MSCs to calibrate therapeutic capacity of PSC-MSCs is considered a weak point of previous research, which needs to be realized and carefully considered in further studies on the therapeutic validation of derived PSC-MSCs. Additionally, rigorous transcriptome and proteomic analyses of the derived PSC-MSCs are needed to reveal key factors associated with the unique properties of PSC-MSCs.

The efficacy of MSCs to migrate to various cancers enables them to be exploited as a biological tool for the delivery of the anti-cancer compounds [180]. However, MSCs are notorious for their induction of tumor growth and metastasis [181]. Moreover, the potency of PSC-MSCs to migrate to cancer sites has been shown; yet, PSC-MSCs possessed less tumorigenicity action than adult MSCs, despite the tumorigenic nature of their original cells (PSCs) [182]. The low expression level of receptors for pro-tumor factors and interleukins compared with MSCs is the main attribute involved in this unique action of PSC-MSCs over primary MSCs [148].

iPSCs used for MSC derivation have a wide range of biosafety issues. For instance, the generation of iPSCs is carried out using integrative viral-based methods or the oncogenic c-Myc for somatic cell reprogramming, which can lead to tumor formation when injected into immunocompromised mice [183]. Previous reports have shown that iPSC-MSCs did not show tumorigenicity, although they were derived from iPSCs [132,135]. However, any remnants of the original iPSCs in the differentiated MSCs can be a major biosafety issue as they will induce tumor formation when applied in immunocompromised patients. Therefore, this issue should be seriously taken into account in the future. iPSCs assigned for differentiation into MSCs should be generated using methods that eliminate integrative viral-based methods and should be cultured under xenogeneic-free culture conditions. In addition, stringent analysis to assure the absence of any residual iPSCs in the differentiated MSCs needs to be considered in the future.

The administration of PSC-MSCs into animal disease models showed differential actions based on the administration route [32], which requires further characterization. The best route for cell administration needs to be characterized. Moreover, the detailed biological journey of the in vivo transplanted PSC-MSCs from administration-to-release needs further investigations.

**Figure 8:**
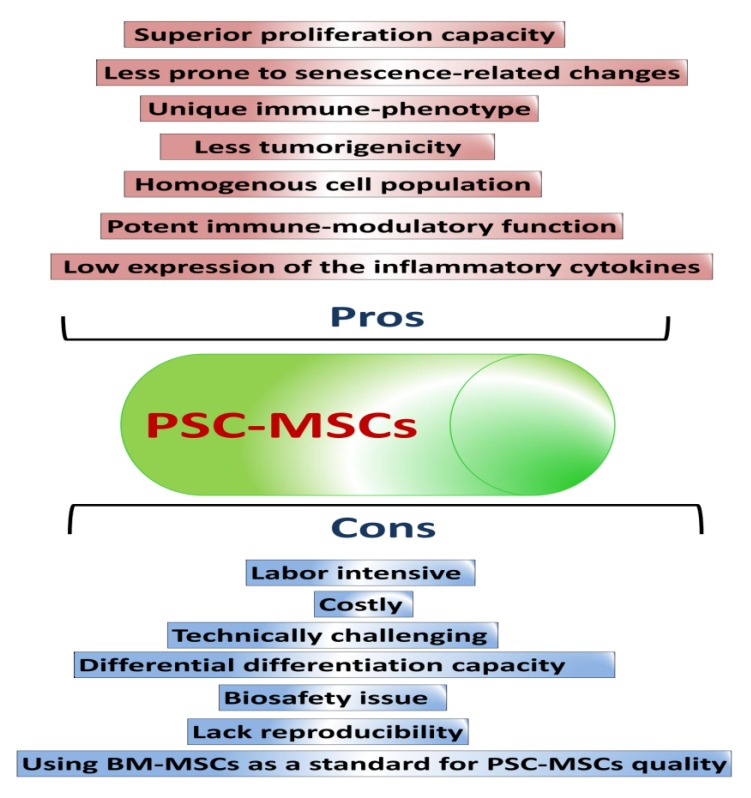
Representative diagram summarizing the pros and cons of PSC-MSCs.

PSC-MSCs have shown significantly higher proliferation capacity, stronger immunomodulatory function, and lower senescence-related changes than adult MSCs. In contrast, they possess different tri-lineage differentiation capacity than that shown by adult MSCs, which needs further explanation. For instance, the expression levels of markers related to adipogenic differentiation in PSC-MSCs are lower than those shown in adult MSCs [117,146,155,184,185], whereas the osteogenic differentiation capacity is significantly higher in iPSC-MSCs [100,129,131]. The factors and signaling molecules involved in the difference in differentiation propensity between PSC-MSCs need to be revealed. Further studies on the epigenetic memory of MSCs derived from PSCs need to be performed. There are some controversies on the therapeutic applications of PSC-MSCs, which need further validation. Moreover, some activities of PSC-MSCs have only been shown in vitro; thus, further in vivo verification is essential. The pros and cons of PSC-MSCs production are summarized in Figure 8.

iPSC-MSC-Exo has shown higher or similar therapeutic activity in various diseases. Further research on the large-scale production of iPSC-MSC-Exo for efficient therapy of incurable diseases needs to be rapidly developed.

There is a dearth of studies detailing the signaling molecules implicated in the derivation of MSCs from PSCs, which requires further consideration. In addition, the epigenetic memory of PSC-MSCs and its similarity or variation with the original somatic cells need to be scrutinized.

Taking into account all the aforementioned points between the primary or tissue-derived MSCs and PSC-MSCs will open avenues for further improvement of the derivation methodology of MSCs from PSCs, as well as the characterization and the validation of their therapeutic capacities.

## 5. Conclusions

A great body of literature and clinical trials have shown the therapeutic capacities of MSCs. However, tissue-derived MSCs possess several shortcomings, such as difficult retrieval methods, lack of reproducibility, heterogenic cell population, senescence-related changes, and loss of proliferation and self-renewal capacities over continuous passages. The need for more than one dose of cells for therapeutic application is also one of the main hurdles. PSCs are a potent source for derivation of MSCs. We discussed detailed protocols for PSC-MSC differentiation, which originated from ESCs. Most of the methods are laborious, time-consuming, and costly; however, there have been recent improvements in these protocols. PSC-MSCs have shown superior proliferative capacity, longevity, immunomodulatory function, and therapeutic applications. In particular, PSC-MSC derivation using the 3D platform (spheroid culture) showed enhanced activities and large-scale generation of MSCs. There are variations in the differentiation capacity between PSC-MSCs and primary MSCs that require further investigations. However, in-depth investigations should characterize key biomolecules involved in the potent therapeutic activity of PSC-MSCs. Moreover, efficient, cost-effective, and reproducible protocols need to be created for large-scale production of MSCs with unique therapeutic potentials that far exceed tissue-derived MSCs, which could be a breakthrough in regenerative medicine.

## Figures and Tables

**Table 1:** Generation methods and the therapeutic efficacies of pluripotent stem cells-derived mesenchymal stem cells (PSC-MSCs). SCID—severely combined immunodeficient; hESC—human embryonic stem cells; UC-MSC—umbilical cord-derived MSCs; CD—cluster of differentiation; VEGF—vascular endothelial growth factor; MEF—mouse embryonic fibroblast; CM—conditioned medium; bFGF—basic fibroblast growth factor; HLA—human leukocyte antigen; EBs—embryonic bodies; PDGFR-α—platelet-derived growth factor receptor alpha; ECM—extracellular matrix; SSEA—stage-specific embryonic antigen; BM—bone marrow; EAE—experimental autoimmune encephalitis; IL—interleukin; MCT—monocrotaline; PAH—pulmonary arterial hypertension; BMP—bone morphogenetic protein; DBM—demineralized bone matrix; i.t.—intrathecal; TGF—transforming growth factor; IKK—inhibition of IκB kinase; KCNH1—potassium voltage-gated channel, subfamily H (eag-related), member 1; NF-κB—nuclear factor kappa B; ALP—alkaline phosphatase; hEAG—human ether-à-go-go; EGF—epidermal growth factor; GFP—green fluorescent protein; DOX—doxorubicin; ROS—reactive oxygen species; DC—dendritic cell; PL—platelet lysate; CPC—calcium phosphate cement; PMEDSAH—poly [2-(methacryloyloxy) ethyl dimethyl-(3-sulfopropyl) ammonium hydroxide; NCC—neural crest cells; LNGFR—low-affinity nerve growth factor receptor; THY—thymocyte antigen; NOD/SCID—non-obese diabetic/severe combined immunodeficiency; NK—natural killer.

PSC Lines	Derivation Method	Characteristic Features	Therapeutic Efficacy
ESC-MSC	Co-culture of hESCs with murine OP9 stromal cells (Barberi et al., 2005 [89])	-Spindle-like morphology. -Positively expressed CD105, STRO-1, CD106, CD29, CD44, CD54, CD166, vimentin, and alpha smooth muscle actin and negatively expressed CD34, CD45, and CD14. -Late detection of CD73^+^ cells (at day 40). -Tri-lineage differentiation.	-n.d. *
	Co-culture method of Barberi et al., but with irradiated murine OP9 stromal cells (Trivedi et al., 2007 [91]).	-Positive for CD73, CD29, CD44, CD54, CD90 and, CD105. -Negative for CD34 and CD45. -Early detection of CD73^+^ cells that followed by the emergence of CD34^+^ cells (within first 2 weeks). - Tri-lineage differentiation.	-n.d. *
	Culture on Matrigel plate with MEF-CM+bFGF (Trivedi et al., 2008 [92])	-Positive expression for CD29, CD44, CD54, CD73, 90, and CD105. -Negative expression for CD34, CD45, and CD31 -High expression of HLA class I, but no expression of HLA class II. - Tri-lineage differentiation.	-Inhibited the proliferation of responder T-lymphocytes [92]. -Co-transplantation of hESC-MSCs (conditionally overexpressed with VEGF) with islet resulted in resulted in 50% reduction of the required islet mass in diabetic mice through promoting islet revascularization [119].
	EBs’ formation with gelatin coating and mechanical scraping (Hwang et al., 2008 [93])	-Fibroblast-like morphology. -Positive expression for CD13, CD14, CD29, CD44, CD73, CD90, CD105, CD146, CD166, STRO-1, and PDGFR-α. -Negative expression for CD34, CD45, CD117, and CD133. -Tri-lineage differentiation.	-New cartilage formation (rich in ECM) upon transplantation into the athymic mice for 12 weeks. -Cartilage defects complete recovery after the transplantation in the articular cartilage defects in the femoral condyle of athymic nude rats.
	EBs formation with gelatin coating + bFGF (Brown et al., 2009 [94])	-Similar characteristics to hBM-MSCs with a higher proliferative capacity. -Positive for CD73 and STRO-1, and lacked the expression of CD45. -Osteogenic and adipogenic differentiations.	-In vitro generation of osteoprogenitor cells after the transduction with bone-associated lentiviral Col2.3-GFP.
	Repeated passage with trypsinization with MSC culture medium (Yen et al., 2011 [95])	-Similar characteristics to hBM-MSCs. -Positively expressed CD29, CD44, CD73, CD90, and CD105, whereas negatively expressed CD14, CD34, and CD45. -Weak expression of SSEA-4 as well as CD9 and no expression of TRA-1-60 or TRA-1-81. -No teratoma formation when injected in immune-compromised mice. -Tri-lineage differentiation.	-Highly expressed geneses associated with transcriptional and proliferative processes (Transcriptome profiling analysis)
	Hemangioblast: Bi-potential progenitors derived from EBs (using cytokine-rich media) (Kimbrel et al., 2014 [26])	-Similar characteristics to BM-MSCs with a higher proliferative capacity and smaller size. -Higher expression of CD10 and CD24 than that of adult BM-MSCs. -Tri-lineage differentiation.	-Suppression of dendritic cell-associated high production of IL-12p70 and the high level of CD83 [26]. -Therapeutic activity against autoimmune disorder mouse models, such as EAE and lupus nephritis and uveitis [26]. -Showed a superior activity than that of BM-MSCs in against EAE mouse model and the neuronal demyelination that attributed to the low expression of IL-6 [98].
	Defined culture condition-based method with PDGF-AB, and bFGF (Lian et al., 2007 [99])	-Similar characteristics to BM-MSCs. -Reduced expression of pluripotency-related genes (*HESX1*, *POUFL5*, *SOX-2*, *UTF-1*, and *ZFP42*) and decreased protein level of OCT4 and SOX2. -Positive expression for CD29, CD44, CD49a, CD105, and CD166. -Negative expression for CD34 and CD45. -No teratoma formation 4 months post-transplantation. -Tri-lineage differentiation.	-Efficient therapeutic activity against MCT-induced PAH mouse model [115].
	Defined culture condition-based method (using xeno-free hESCs and culture conditions) (Karlsson et al., 2009 [100])	-Fibroblast-like morphology. -Loss of expression of Oct-4, Nanog, TRA 1-60, TRA 1-81, SSEA-3, and SSEA-4. -Loss of expression of the endoderm- and neuroectoderm-related markers. -Positive for CD105, CD166, CD10, and CD13, whereas negative for CD133 and CD117. -Tri-lineage differentiation.	-Transplantation into SCID mice resulted in formation of well-defined tissues of MSC origin without teratoma formation.
	Trophoblast-like stage (With BMP4 and activin-like receptor kinases inhibitor) (Wang et al., 2016 [106])	-Downregulation of trophoblast-related genes (from day 11 to day 16). -Positive for CD73, CD90, CD105, CD29, and CD44. -Negative for Trop2, CD31, and CD34. -Tri-lineage differentiation.	-In vitro and in vivo immunomodulatory activity.
	3D platform (formation of trophoblast-like stage in spheroid) (Yan et al., 2018 [32])	On day 10: Positive expression for trophoblast- and MSC-related markers. -On day 20: No detection of trophoblast-associated markers and upregulation of MSC-related markers. -Positive for CD90, CD105, and CD44. -Negative for apoptotic markers. -Tri-lineage differentiation.	-Potentially adhered and differentiated into bone and cartilage in DBM scaffold [32]. -Potent in vitro immunomodulatory activity [32]. -Robust therapeutic activity in mouse model of inflammatory colitis [32]. -Recovery of multiple sclerosis using EAE model in cynomolgus monkeys through the i.t. injection [120].
	Small molecule inhibitors (TGF-β/activin/nodal signaling pathway inhibitor, SB-431542 (Mahmood et al., 2010 [112])	-Positive for CD44, CD73, CD146, and CD166. -Downregulation of myogenesis-related genes.	-In vitro and in vivo tri-lineage differentiation capacities.
	Small molecule inhibitors (SMAD-2/3 signaling pathway inhibitor) (Sanchez et al., 2011 [113])	-Positive expression for CD73 and CD90, whereas negative for CD34 expression. -Low expression of the pluripotency markers (Oct4 and Tra-1-60). -Tri-lineage differentiation. -Higher proliferation capacity than BM-MSCs.	-Potent in vivo anti-inflammatory and immunosuppressive activities in a mouse model of experimental colitis. -Alleviation of collagen-mediated arthritis in mice through the upregulation of the expression of IDO1 [118].
	Small molecule inhibitors (IKK/NF-κB signaling inhibitor) (Deng et al., 2016 [114])	-Loss pluripotency markers expression level. -Decrease in ALP activity. -Positive expression for CD51 and CD90, whereas negative for CD34 and CD45.	-In vitro and in vivo bone formation.
	Small molecule inhibitors (TGF-β pathway inhibitor, SB431542) (Chen et al., 2012 [117])	-MSC-like morphology. -Positive for the expression of CD105, CD73, and CD90, whereas lacked the expression of CD45 and CD14. -Marked reduction in the expression level of the pluripotency-associated markers.	-Potent neuroprotective effect in a hypoxic-ischemic mouse brain model and better than that shown by fetal MSCs [116].
iPSC-MSC	Defined culture conditions with growth factors bFGF, EGF, and PDGF-AB (Lian et al., 2010 [122])	-Similar to BM-MSCs with higher proliferation capacity. -Positive for CD44, CD49a, CD73, CD105, and CD166. -Negative for CD34, CD45, and CD133. -Tri-lineage differentiation.	-Therapeutic activity against severe hind-limb ischemia mouse model [122]. -Increased hEAG1 potassium channel encoded by KCNH1 [124]. -Robust immunomodulatory function through marked reduction of phytohaemagglutinin-induced lymphocyte proliferation as well as decreased the proliferation of CD3-positive T cells [164]. -Alleviation of cigarette smoke-related pulmonary damage in rat model via the mitochondrial transfer mechanism [167]. -In vitro and in vivo attenuation of DOX-mediated cardiomyopathy via reduction of ROS generation [162]. -Suppression the early stage differentiation of CD14^+^ monocytes to DCs and blocked DC-mediated T cell activation [150]. -Combination with the low concentration of rapamycin markedly increased the survival rate of the islet allograft in the diabetic mice [161].
	Repeated passage with trypsinization with MSC culture medium (Zou et al., 2013 [125])	-Fibroblast-like morphology. -Positive expression for CD90, CD73, and CD105. -Loss of the expression of pluripotency markers (OCT3/4 and TRA-1-81), whereas still positive for Nanog -Tri-lineage differentiation.	-In vitro osteogenic differentiation.
	Hypoxic condition with growth factor (Yang et al., 2014 [128])	-Similar to rat BM-MSCs. -Positively expressed CD29 and CD90, whereas negatively expressed CD34 and CD45.	-In vivo anti-inflammatory activity using a rat model of experimental periodontitis.
	Using PL supplement (Luzzani et al., 2015 [129])	-Share characteristics with the UC-MSC. -Positive for CD90, CD73, CD105, CD166, and CD271. -Tri-lineage differentiation.	-In vitro immunomodulating activity through the suppression of concanavalin-A-induced lymphocyte proliferation.
	Biomimetic, fibrillar, type I collagen coatings (Liu et al., 2012 [131]	-Positive expression for CD90, CD105, CD166, CD73, and CD146, whereas negative for CD34 and CD45. -Tri-lineage differentiation. -Spindle-shaped morphology.	-n.d. *
	PMEDSAH coating (Villa Diaz et al., 2012 [132])	-Positively expressed CD44, CD73, CD105, and CD166, whereas lacked the expression of CD34 and CD45. -Tri-lineage differentiation.	*-*In vivo bone regenerative capacity in calvaria defects mouse model.
	EB formation with gelatin coating (Tang et al., 2014 [136])	-Positive expression of MSC markers. -Lacked the expression of the hematopoietic markers and the pluripotency markers. -Tri-lineage differentiation.	-Efficient in vitro osteogenic differentiation in CPC scaffold.
	Small molecule inhibitors (TGF-β pathway inhibitor, SB431542) (Chen et al., 2012 [117])	-MSC-like morphology. -Decrease in the pluripotency markers. -Tri-lineage differentiation. -No in vivo teratoma formation. -High expression of vimentin and N-cadherin.	-Potent neuroprotective effect in a hypoxic-ischemic mouse brain model and better than that shown by fetal MSCs [116].
	Gelatin coating (Hyunes et al., 2013 [137])	-Fibroblastic-like morphology. -Positive expression for CD73, CD90, CD105, CD146, and CD166. -Lacked the expression of the pluripotency markers. -Negative expression for CD14, CD34, and CD45. -Tri-lineage differentiation.	-In vitro immunomodulating activity through the suppression of concanavalin-A-induced mouse splenocyte proliferation [165]. -Anti-inflammatory activity in mouse model implanted with *P. gingivalis* containing sponge [165].
	EB formation with poly-hydroxyethyl methacrylate and gelatin coatings (Sheyn et al., 2016 [138])	-Similar characteristics to BM-MSCs. -Tri-lineage differentiation.	-In vivo bone formation.
	Using NCCs (Ouchi et al., 2016 [140])	-Spindle-like morphology. - High expression of LNGFR and THY-1. -Differentiation into neural crest-related cells.	-High proliferative capacity upon transplantation into chicken embryo and can migrate to the sclerotome region [140]. -Recovery of the peripheral nerve injury in NOD/SCID mice with sciatic nerve injury [144].
	MSC culture medium supplemented with bFGF (Giuliani et al., 2011 [145])	-Spindle-shaped morphology. -High expression of CD90, CD105, CD146, CD54, and CD73. -Lacked the expression of CD45), HLA class II (HLA-DR), and costimulatory molecules. -No expression of the pluripotency factors	-Potent superior immunomodulatory activity than of BM-MSCs and after various passages. -Decreased NK proliferation and its cytolytic property.
	Redifferentiation of iPSC reprogrammed from the reprogramming of BM-MSCs (Frobel et al., 2014 [146])	-MSC-like morphology. -Tri-lineage differentiation. -Positive expression for CD29, CD73, CD90, and CD105 (less expression), whereas negative expression for CD14, CD31, CD34, and CD45.	-Immunomodulatory function, but lower than the original MSCs.
	Small molecule inhibitors (SMAD-2/3 inhibitor, SB-431542)(Zhao et al., 2015 [148])	-Spindle-like morphology. -Positive expression for CD73, CD105, CD166, CD44 and CD90), whereas negative for HLA-DR, CD11b, CD24, CD34, and CD45. -High expression of mesodermal markers CD140A*/*PDGFRα -Significant decrease in the expression of the pluripotency factors and the neuroectodermal factors.	-Potent in vivo tumor homing activity similar to that of BM-MSCs, whereas with lower pro-tumor activity than BM-MSCs and thus avoiding tumor progression.
	Commercially purchased iPSC-MSCs derived from fetal and adult BM (Sun et al., 2015 [149])	-Positive expression for CD44, CD105, CD90, and CD73, whereas lacked the expression of CD45, CD14, CD34, CD3, and CD56. -High expression level of of HLA class I.	-Superior effect in the attenuation of the in vivo inflammation in induced hind limb ischemia mouse model than that of BM-MSCs.
	EB formation method (With MSCs differentiation medium + all trans retinoic acid and the continuous passage for 4 months (Himeno et al., 2013 [160])	-Positively expressed CD105, CD140a, Sca-1, and CD44. -Negatively expressed CD34, TER119, CD31, CD45, and CD11b. -Tri-lineage differentiation.	-In vivo attenuation of diabetes-related polyneuropathy in streptozotocin-diabetic mice.

* n.d., not determined.

## Abbreviations

AD-MSCs	Adipose-Derived MSCs
ALP	Alkaline Phosphatase
BEAS-2B	Bronchial Epithelial Cells
BM-MSCs	Bone Marrow-Derived MSCs
bFGF	Basic Fibroblast Growth Factor
CD	Cluster of Differentiation
CFU-F	Colony Forming Unit Fibroblasts
CM	Conditioned Medium
CNS	Central Nervous System
CPC	Calcium Phosphate Cement
DBM	Demineralized Bone Matrix
DCs	Dendritic Cells
DMEM	Dulbecco’s Modified Eagle’s Medium
DOX	Doxorubicin
ECM	Extracellular Matrix
EAE	Experimental Autoimmune Encephalitis
EBs	Embryonic Bodies
EMT	Epithelial-to-Mesenchymal Transition
FACS	Fluorescence-Activated Cell Sorting
FBS	Fetal Bovine Serum
GDF	Growth/Differentiation Factor
hESC-MSC_SP_	Spheroidal hESC-MSC
HLA	Human Leukocyte Antigen
IDO1	Indoleamine 2,3 Dioxygenase
IFNγ	Interferon γ
IKK	IκB Kinase
i.m.	Intramuscular
ISCT	International Society for Cellular Therapy
i.v.	Intravenous
KO-SR	Knockout Serum Replacement
LEF	Lymphoid Enhancer-Binding Factor
LNGFR	Low-Affinity Nerve Growth Factor Receptor
MCT	Monocrotaline
MEF	Mouse Embryonic Fibroblast
MIF	Macrophage Inhibitory Factor
MPs	Mesenchymal Progenitors
MSCs	Mesenchymal Stem Cells
NCC	Neural Crest Cells
NCLCs	NC-Like Cells
NF-κB	Nuclear Factor Kappa B
NK cells	Natural Killer Cells
OA	Osteoarthritis
PAH	Pulmonary Arterial Hypertension
PDGFRa	Platelet-Derived Growth Factor Receptor Alpha
PEGD	PEG-Diacrylate
PL	Platelet Lysate
PMEDSAH	Poly [2-(methacryloyloxy) Ethyl Dimethyl-(3-Sulfopropyl) Ammonium Hydroxide
PBMCs	Peripheral Blood Mononuclear Cells
ROCK	Rho-Associated, Coiled-Coil Containing Protein Kinase
ROS	Reactive Oxygen Species
SSEA-4	Stage-Specific Embryonic Antigen-4
s.c.	Subcutaneous
TGF-β1	Transforming Growth Factor-Beta 1
THY-1	Thymocyte Antigen-1
T-MSC	Trophoblast-derived Mesenchymal Stem Cells
TNFα	Tumor Necrosis Factor-Alpha
TSG	Tumor Necrosis Factor Alpha-Stimulated Gene
VW	Vascular Wall
VEGF	Vascular Endothelial Growth Factor
